# Supplementary material for: ABVENTURE-P pilot trial of physical therapy versus standard of care following ventral hernia repair: Protocol for a randomized controlled trial
Source: PLoS One. 2023 Jul 27;18(7):e0289038. doi: 10.1371/journal.pone.0289038 (PMC10374060; doi:10.1371/journal.pone.0289038)
Supplement: S1 Appendix — The protocol includes sample exercises, tips for physical therapists to use to elicit proper technique, optimal dosage parameters, and criteria for progression or regression. (PPTX) [file pone.0289038.s001.pptx]

## Slide 1
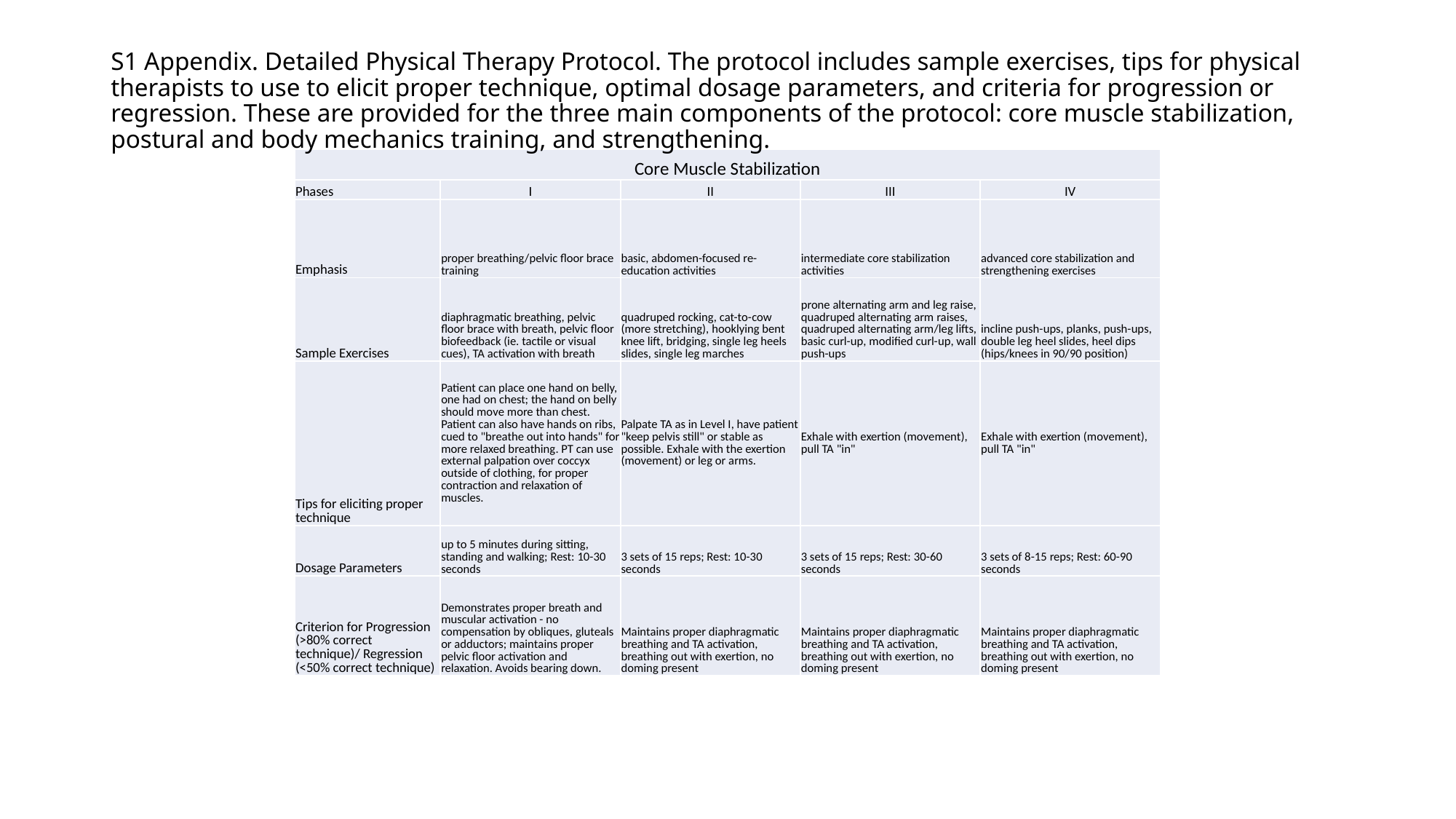

# S1 Appendix. Detailed Physical Therapy Protocol. The protocol includes sample exercises, tips for physical therapists to use to elicit proper technique, optimal dosage parameters, and criteria for progression or regression. These are provided for the three main components of the protocol: core muscle stabilization, postural and body mechanics training, and strengthening.
| Core Muscle Stabilization | | | | |
| --- | --- | --- | --- | --- |
| Phases | I | II | III | IV |
| Emphasis | proper breathing/pelvic floor brace training | basic, abdomen-focused re-education activities | intermediate core stabilization activities | advanced core stabilization and strengthening exercises |
| Sample Exercises | diaphragmatic breathing, pelvic floor brace with breath, pelvic floor biofeedback (ie. tactile or visual cues), TA activation with breath | quadruped rocking, cat-to-cow (more stretching), hooklying bent knee lift, bridging, single leg heels slides, single leg marches | prone alternating arm and leg raise, quadruped alternating arm raises, quadruped alternating arm/leg lifts, basic curl-up, modified curl-up, wall push-ups | incline push-ups, planks, push-ups, double leg heel slides, heel dips (hips/knees in 90/90 position) |
| Tips for eliciting proper technique | Patient can place one hand on belly, one had on chest; the hand on belly should move more than chest. Patient can also have hands on ribs, cued to "breathe out into hands" for more relaxed breathing. PT can use external palpation over coccyx outside of clothing, for proper contraction and relaxation of muscles. | Palpate TA as in Level I, have patient "keep pelvis still" or stable as possible. Exhale with the exertion (movement) or leg or arms. | Exhale with exertion (movement), pull TA "in" | Exhale with exertion (movement), pull TA "in" |
| Dosage Parameters | up to 5 minutes during sitting, standing and walking; Rest: 10-30 seconds | 3 sets of 15 reps; Rest: 10-30 seconds | 3 sets of 15 reps; Rest: 30-60 seconds | 3 sets of 8-15 reps; Rest: 60-90 seconds |
| Criterion for Progression (>80% correct technique)/ Regression (<50% correct technique) | Demonstrates proper breath and muscular activation - no compensation by obliques, gluteals or adductors; maintains proper pelvic floor activation and relaxation. Avoids bearing down. | Maintains proper diaphragmatic breathing and TA activation, breathing out with exertion, no doming present | Maintains proper diaphragmatic breathing and TA activation, breathing out with exertion, no doming present | Maintains proper diaphragmatic breathing and TA activation, breathing out with exertion, no doming present |

## Slide 2
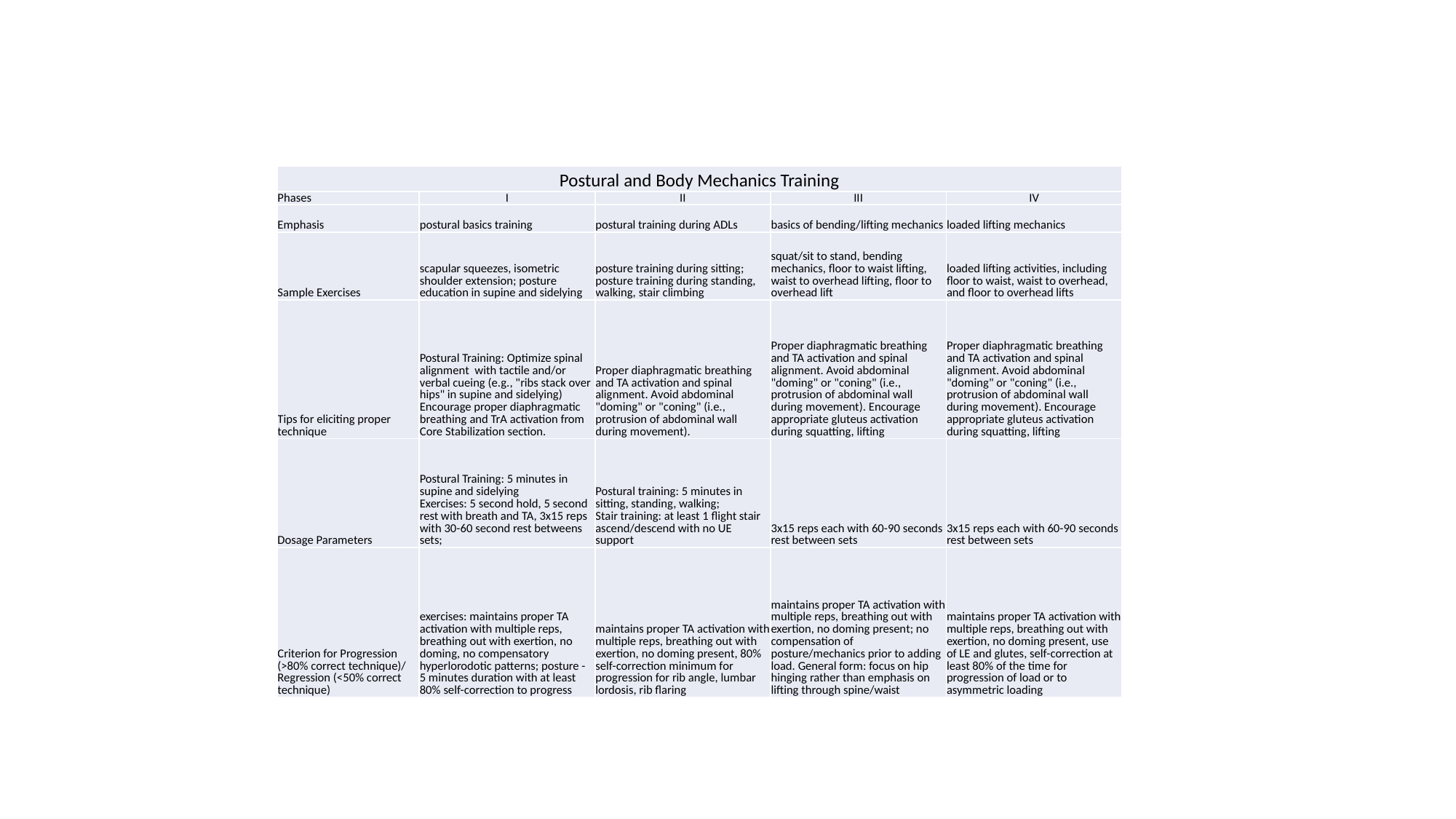

| Postural and Body Mechanics Training | | | | |
| --- | --- | --- | --- | --- |
| Phases | I | II | III | IV |
| Emphasis | postural basics training | postural training during ADLs | basics of bending/lifting mechanics | loaded lifting mechanics |
| Sample Exercises | scapular squeezes, isometric shoulder extension; posture education in supine and sidelying | posture training during sitting; posture training during standing, walking, stair climbing | squat/sit to stand, bending mechanics, floor to waist lifting, waist to overhead lifting, floor to overhead lift | loaded lifting activities, including floor to waist, waist to overhead, and floor to overhead lifts |
| Tips for eliciting proper technique | Postural Training: Optimize spinal alignment with tactile and/or verbal cueing (e.g., "ribs stack over hips" in supine and sidelying) Encourage proper diaphragmatic breathing and TrA activation from Core Stabilization section. | Proper diaphragmatic breathing and TA activation and spinal alignment. Avoid abdominal "doming" or "coning" (i.e., protrusion of abdominal wall during movement). | Proper diaphragmatic breathing and TA activation and spinal alignment. Avoid abdominal "doming" or "coning" (i.e., protrusion of abdominal wall during movement). Encourage appropriate gluteus activation during squatting, lifting | Proper diaphragmatic breathing and TA activation and spinal alignment. Avoid abdominal "doming" or "coning" (i.e., protrusion of abdominal wall during movement). Encourage appropriate gluteus activation during squatting, lifting |
| Dosage Parameters | Postural Training: 5 minutes in supine and sidelying Exercises: 5 second hold, 5 second rest with breath and TA, 3x15 reps with 30-60 second rest betweens sets; | Postural training: 5 minutes in sitting, standing, walking; Stair training: at least 1 flight stair ascend/descend with no UE support | 3x15 reps each with 60-90 seconds rest between sets | 3x15 reps each with 60-90 seconds rest between sets |
| Criterion for Progression (>80% correct technique)/ Regression (<50% correct technique) | exercises: maintains proper TA activation with multiple reps, breathing out with exertion, no doming, no compensatory hyperlorodotic patterns; posture - 5 minutes duration with at least 80% self-correction to progress | maintains proper TA activation with multiple reps, breathing out with exertion, no doming present, 80% self-correction minimum for progression for rib angle, lumbar lordosis, rib flaring | maintains proper TA activation with multiple reps, breathing out with exertion, no doming present; no compensation of posture/mechanics prior to adding load. General form: focus on hip hinging rather than emphasis on lifting through spine/waist | maintains proper TA activation with multiple reps, breathing out with exertion, no doming present, use of LE and glutes, self-correction at least 80% of the time for progression of load or to asymmetric loading |

## Slide 3
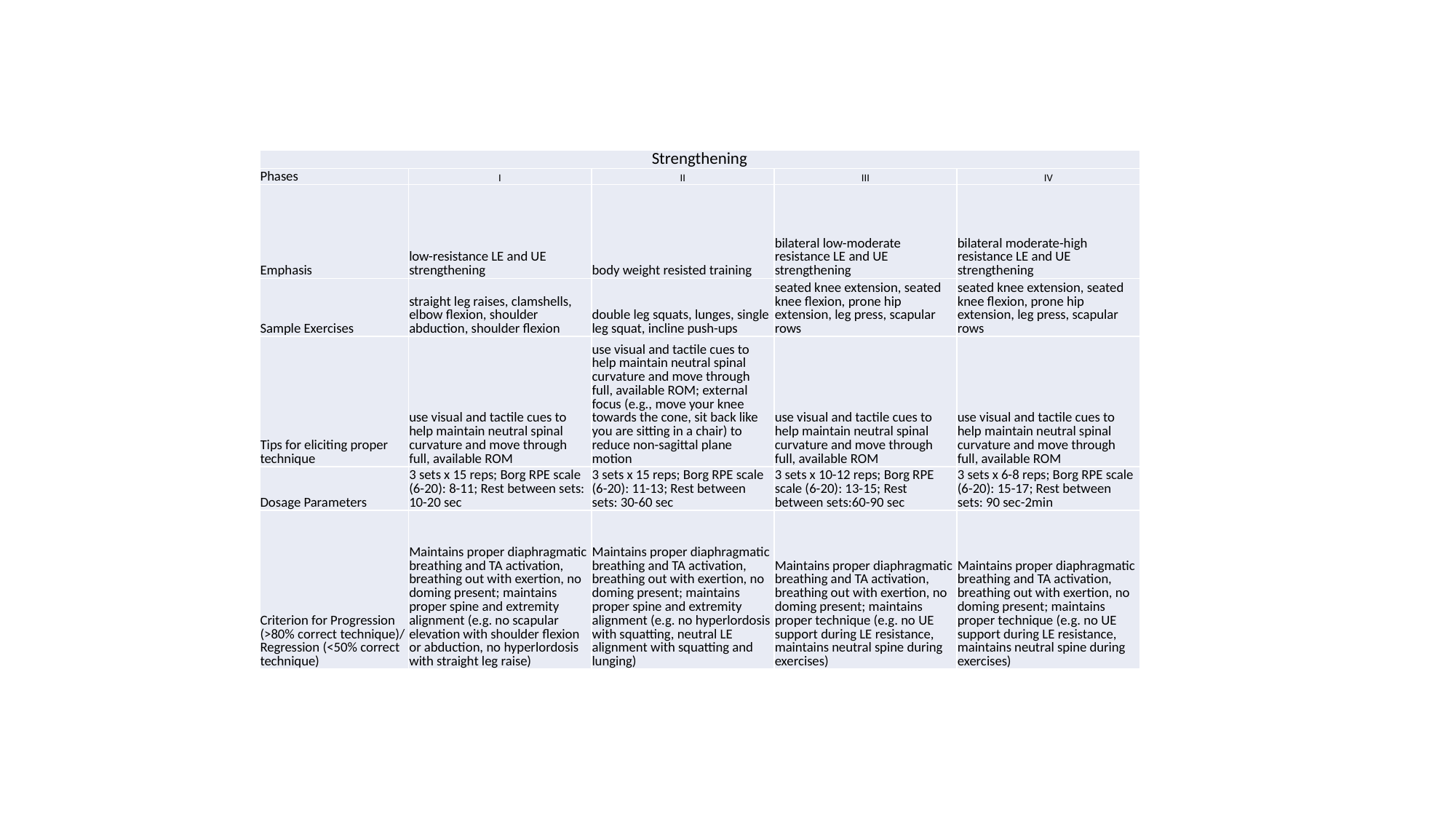

| Strengthening | | | | |
| --- | --- | --- | --- | --- |
| Phases | I | II | III | IV |
| Emphasis | low-resistance LE and UE strengthening | body weight resisted training | bilateral low-moderate resistance LE and UE strengthening | bilateral moderate-high resistance LE and UE strengthening |
| Sample Exercises | straight leg raises, clamshells, elbow flexion, shoulder abduction, shoulder flexion | double leg squats, lunges, single leg squat, incline push-ups | seated knee extension, seated knee flexion, prone hip extension, leg press, scapular rows | seated knee extension, seated knee flexion, prone hip extension, leg press, scapular rows |
| Tips for eliciting proper technique | use visual and tactile cues to help maintain neutral spinal curvature and move through full, available ROM | use visual and tactile cues to help maintain neutral spinal curvature and move through full, available ROM; external focus (e.g., move your knee towards the cone, sit back like you are sitting in a chair) to reduce non-sagittal plane motion | use visual and tactile cues to help maintain neutral spinal curvature and move through full, available ROM | use visual and tactile cues to help maintain neutral spinal curvature and move through full, available ROM |
| Dosage Parameters | 3 sets x 15 reps; Borg RPE scale (6-20): 8-11; Rest between sets: 10-20 sec | 3 sets x 15 reps; Borg RPE scale (6-20): 11-13; Rest between sets: 30-60 sec | 3 sets x 10-12 reps; Borg RPE scale (6-20): 13-15; Rest between sets:60-90 sec | 3 sets x 6-8 reps; Borg RPE scale (6-20): 15-17; Rest between sets: 90 sec-2min |
| Criterion for Progression (>80% correct technique)/ Regression (<50% correct technique) | Maintains proper diaphragmatic breathing and TA activation, breathing out with exertion, no doming present; maintains proper spine and extremity alignment (e.g. no scapular elevation with shoulder flexion or abduction, no hyperlordosis with straight leg raise) | Maintains proper diaphragmatic breathing and TA activation, breathing out with exertion, no doming present; maintains proper spine and extremity alignment (e.g. no hyperlordosis with squatting, neutral LE alignment with squatting and lunging) | Maintains proper diaphragmatic breathing and TA activation, breathing out with exertion, no doming present; maintains proper technique (e.g. no UE support during LE resistance, maintains neutral spine during exercises) | Maintains proper diaphragmatic breathing and TA activation, breathing out with exertion, no doming present; maintains proper technique (e.g. no UE support during LE resistance, maintains neutral spine during exercises) |
