# Supplementary material for: ABVENTURE-P pilot trial of physical therapy versus standard of care following ventral hernia repair: Protocol for a randomized controlled trial
Source: PLoS One. 2023 Jul 27;18(7):e0289038. doi: 10.1371/journal.pone.0289038 (PMC10374060; doi:10.1371/journal.pone.0289038)
Supplement: S1 Data — (DOCX) [file pone.0289038.s003.docx]

**ABVENTURE-P: Pilot Trial of Abdominal Core Rehabilitation To Improve Outcomes**

**After Ventral Hernia Repair**

**Co-Principal Investigators:**

Ajit Chaudhari, PhD

Ohio State University

School of Health & Rehabilitation Sciences

[Ajit.chaudhari@osumc.edu](mailto:Ajit.chaudhari@osumc.edu)

Benjamin Poulose, MD, MPH

Ohio State University Wexner Medical Center

Department of Surgery

Division of General and GI Surgery

[Benjamin.poulose@osumc.edu](mailto:Benjamin.poulose@osumc.edu)

Stephanie Di Stasi, PhD

Ohio State University

School of Health and Rehabilitation Sciences

Division of Physical Therapy

[Stephanie.distasi@osumc.edu](mailto:Stephanie.distasi@osumc.edu)

#
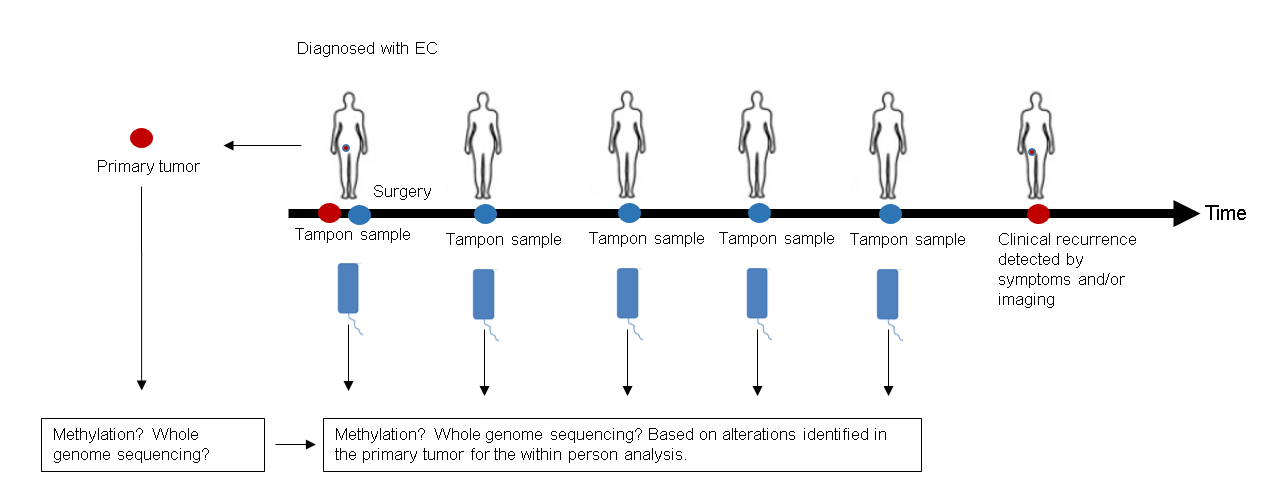
SPECIFIC AIMS

Hernia disease is one of the most common ailments that disrupts the function of the abdominal core, with annual surgical repairs nearing 350,000 in the United States alone.^1^ The abdominal core, which consists of the anterior abdominal wall, spine, flanks, diaphragm, and pelvic floor, supports multiple critical functions important to activities of daily living, from respiration to personal hygiene to independent mobility. We define abdominal core health as encompassing the function, stability, and quality of life involving the abdominal core. The current lack of understanding of how hernia disease negatively impacts both abdominal core health and patient well-being precludes the development of effective treatment. Our preliminary work indicates that abdominal core dysfunction is related to patient-reported disability, and highlights new potential targets for exercise-based intervention.

**We propose that the most effective, patient-centered treatment approach for patients undergoing ventral hernia repair requires rehabilitation of the abdominal core.** Standards of practice for many common orthopaedic conditions (e.g. joint replacement) now include physical rehabilitation to maximize beneficial outcomes after surgery. Over recent years, the concept of the abdominal core as a dynamic, functional unit has increasingly gained acceptance among medical professionals treating hernia disease.^2^ However, this recognition has not yet led to inclusion of physical rehabilitation as the standard of care following hernia surgery. Therefore, there is a critical need to establish (a) whether post-operative rehabilitation can augment outcomes of hernia repair surgery, and (b) how disruptions to and changes in abdominal core function over time affect patient outcomes.

Our long-term goal is to develop therapies to improve function of the abdominal core musculature and to regain quality of life considering the abdominal core’s inter-related components. As a first step toward this goal, we have developed an evidence-based post-operative physical therapy (PT) protocol to improve core stability and other contributors to function.^3^ We also propose an innovative and practical way to assess abdominal core function using a Quiet Unstable Sitting Test (QUeST) that we have developed in previous NIH-funded research (R03AR065215).^4^ The feasibility of our approach is supported by preliminary data demonstrating lower QUeST core function scores in hernia patients compared to patients without hernia. Our central hypothesis is that patients undergoing a specific type of hernia repair, ventral hernia repair, will have measurably improved functional performance and quality of life following PT that addresses the mechanism of both loss and gain of abdominal core function relative to the standard of care (post-operative precautions). This proposal directly addresses PAS-20-160, which encourages “**pilot and feasibility clinical trials** conducted in humans that will lay the foundation for larger clinical trials related to the prevention and/or treatment of diseases and conditions within the mission of NIDDK...[to] acquire preliminary data regarding the effects of the intervention, as well as feasibility data related to recruitment and retention, and study conduct.... **Preliminary data regarding intervention efficacy are not require**d.”^5^ We propose a pilot RCT with the following Aims:

**Aim 1:** Establish the efficacy of standardized post-operative PT to improve function and patient-reported out-comes after ventral hernia repair. We hypothesize that patients randomized to a standardized PT protocol will achieve larger gains in function and patient-reported outcomes than those randomized to standard-of-care activity limitations. This registry-based RCT will leverage data captured for patients undergoing hernia repair within the Abdominal Core Health Quality Collaborative along with additional measures added for this study. A standardized PT protocol^3^ will be performed, with function assessed using the Five Times Sit-to-Stand, and patient-reported outcomes using the PROMIS-Physical Function survey. Our primary endpoint is 10 weeks post-op at the conclusion of rehabilitation, with additional time points at 30 days, 6 months and 1 year post-op.

**Aim 2:** Establish the feasibility of this registry-based RCT protocol to test the effect of PT to improve functional and clinical outcomes in individuals undergoing ventral hernia repair. We hypothesize that retention rates (as measured by participation in testing at 10 weeks post-op) will be >80% in both treatment groups, and that compliance rates (as measured by session attendance) will be >80% in the PT group. We anticipate the rehabilitation and testing protocols will be well tolerated by all participants.

**Aim 3:** Determine whether baseline abdominal core function influences the efficacy of post-operative physical therapy after ventral hernia repair. We hypothesize that those in the PT group with poorer core function at enrollment will achieve greater improvements in objective and self-reported physical function from enrollment to 10 weeks post-op than those with better core function. We will use our novel quantitative, continuous abdominal core function assessment (QUeST) at primary and intermediate time points. Our preliminary data demonstrate that the QUeST is reliable. They also show that hernia patients awaiting surgery have significantly worse QUeST scores than people without hernia disease, and there is wide variation in QUeST score among hernia patients, from being within normal limits to three or more standard deviations away from normal.

## Innovation

Traditional approaches to disease management are increasingly compartmentalized, reflected in the development of narrow sub-specialties, especially in surgical care. One surgeon may have expertise in the anterior abdominal wall, another in the pelvic floor, and yet another in diseases of the diaphragm. This approach allows for the successful diagnosis and treatment of these individual components, but it fails to consider the interrelated-ness of these components to function as a holistic unit to improve health. Recent innovations and quality-improvement efforts have reflected these siloed approaches from surgical sub-specialties to “fix” their surgical techniques and materials, and therefore have not included other health care providers whose insights and treatment approaches are directly related to abdominal core health. Moreover, the within-specialty approach to innovation has created a blind spot in terms of understanding and appreciating how the components of the abdominal core inter-relate to one another. While attempts are being made to improve the integrity of mesh repair devices and prevent surgical complications, these do not address the larger functional and self-reported problems that exist in patients regardless of whether repair failure occurs. Lastly, standard patient-centered outcome measures have been limited to self-reports that are not sensitive to change, not quantitative, and not objective.

The proposed research is innovative because it departs from the status quo by breaking down silos of expertise and structures examined, and leverages diverse and complementary perspectives in a holistic manner. First, we are establishing a new paradigm of thought in the management of diseases affecting abdominal core health, which will in turn allow new breakthroughs to improve the quality of life for patients. Second, physical therapists are trained in the prescription of exercise to optimize function and physical activity participation. Through target-ed exercise, physical therapy can restore muscle performance, eliminate pain and enable healthy movement in individuals with hernia disease. For the first time, we include physical therapy with the standard surgical care for these patients to fill the current treatment gaps and significantly improve patients’ quality of life. Lastly, we intro-duce quantitative, objective, and sensitive measures to the field of Hernia Repair and Recovery that assess the functional integration of the abdominal core in a clinically feasible way. The design and execution of this pilot RCT will support a future multi-center RCT to improve outcomes in this patient population, which will in turn al-low new breakthroughs to improve the quality of life for patients. Second, physical therapists are trained in the prescription of exercise to optimize function and physical activity participation. Through targeted exercise, physical therapy can restore muscle performance, eliminate pain and enable healthy movement in individuals with hernia disease. For the first time, we include physical therapy with the standard surgical care for these patients to fill the current treatment gaps and significantly improve patients’ quality of life. Lastly, we introduce quantitative, objective, and sensitive measures to the field of Hernia Repair and Recovery that assess the functional integration of the abdominal core in a clinically feasible way. The design and execution of this pilot RCT will sup-port a future multi-center RCT to improve outcomes in this patient population.

# BACKGROUND AND RATIONALE

Hernia disease is one of the most common reasons patients seek surgical treatment, yet outcomes have not improved over time. Over 350,000 ventral hernia repairs (VHRs) are performed yearly in the United States,^1,6^ with an estimated healthcare system cost of more than $4.9 billion.^1^ Recurrence rates for VHR are as high as 32% within 10 years,^7–9^ and are linked to the development of chronic pain.10 Meshes, which are prosthetic medical devices implanted into patients as part of the repair, can reduce the chance of recurrence, but carry additional risk for pain, infection, adhesions, bowel obstruction, mesh migration and ingrowth into vital organs.^8,11^ Importantly, recurrence data alone underestimate the impact of continued pain, which affects one in four individuals post-VHR.^12^ National registry data of more than 7,000 patients undergoing hernia repair and other similar major surgeries indicate 6.5% of patients develop new and persistent opioid use.^13^ Considering the annual number of VHRs performed, up to 32,500 patients post-hernia repair may abuse opioids. Disabling pain-related complications, including problems related to mesh use, have resulted in a ‘perfect storm’ for patients following surgery. These issues, combined with an ineffective post-market surveillance system to monitor performance of mesh products, has led to ‘class action’ lawsuits mainly involving patients against mesh manufacturers. The time, expense, anxiety, and personal toll involved in these efforts may bring justice to some, but will not change the underlying problem in this field of surgery.

Pain, disability and diminished levels of physical activity can persist even when surgery successfully restores the abdominal wall.^14,15^ Our group has found that poor abdominal core function is associated with greater self-reported disability in those awaiting ventral hernia repair (Aim 3 Preliminary Data). The improvement in Quebec Back Pain Disability Scale scores after ventral hernia repair highlights a potential mechanism whereby components of the abdominal core are directly related and can be modulated to improve quality of life.^16^ Due to the protracted length of symptoms and severity of pain experienced, patients with ventral hernia are likely to alter their movement patterns as a strategy to mitigate pain. Like patients with low back pain, the adoption of a ‘stiff’ spine (i.e. less motion) may be an unconscious strategy to reduce pain and protect the vulnerable structures.^17^ These altered movement patterns may provide short-term relief, but are likely to be retained long after the pain re-solves.^17^ Similarly, repair of post-partum diastasis (a problem of the anterior abdominal wall component of the abdominal core) improves self-reported function as measured by the Urogenital Distress Inventory and Incontinence Impact Questionnaire scores.^18^ This study also suggests that inter-relationships between the components of the abdominal core in providing function and quality of life may currently be critically underestimated. Thus, the **goal of this proposal** is to assess the impact of abdominal core stability on post-surgical function and evaluate the feasibility of implementing physical therapy targeted to improving stability and function. Without an understanding of the mechanisms contributing to disability and pain concerning the abdominal core as a holistic and inter-related entity, effective treatments cannot be developed.

Traditionally, management of hernia disease has been limited to surgical restoration of the abdominal wall anatomy, not considering its inter-relatedness to other parts of the body that work together to ensure optimal function. Addressing this problem is a major priority for the National Institute of Diabetes and Digestive and Kidney Dis-eases, which is a sponsor of the Funding Opportunity Announcement (PAS-20-160) to which we are responding. We propose a **paradigm shift** in the current approach to hernia disease. taking into consideration the functional relationships of the anterior abdominal wall, lower back, the diaphragm, and the pelvic floor as part of a holistic ‘abdominal core.’ Abdominal core health encompasses the stability, function, and quality of life involving the abdominal core.^2^ This holistic view of abdominal core includes a conceptual model (Fig. 1) that acknowledges and addresses the factors leading to function and quality of life involving the abdominal core.^2^ As seen in this model, abdominal core stability – or lack thereof – impacts activities of daily living (ADL) and ultimately quality of life. Performance of activities of daily living, from self-care to lifting to work-related tasks, requires proper function of the abdominal core. Function of these structures is affected by neural control patterns, which result from and respond to sensory information, muscle forces, and pain. Deficits in the neural control patterns of the abdominal core have been linked to chronic low back pain^19–25^ and anterior cruciate ligament injury.^26^ Ultimately, separate surgical fields (abdominal wall [hernia] surgery, spine surgery, foregut [diaphragm surgery], pelvic floor surgery) have progressed independently but there has been no effort to link therapies or develop conceptual models reflective of the natural inter-relatedness of these components of the human body. This lack of holistic under-standing has limited the individual progression of these fields, including hernia surgery.

Our approach is focused on physical therapy (PT), because PT can influence all inputs, control patterns and functions of the abdominal core that lead to improved quality of life. Very little research has evaluated the link between chronic pain and disrupted kinematics (i.e. movement patterns) in the VHR population, despite well-described mechanistic links between pain and alteration of these pathways.^27,28^ These issues can lead to kinesiophobia, wherein patients develop a fear of movement due to pain and impairment of function. Kinesiophobia can lead to fear-avoidant behaviors, where the individual often stiffens or alter their movement pattern to complete the same task^29–31^ or avoids the activity altogether.^32–34^ This pattern of adaptation may be perceived as protective in the short-term (e.g. acute pain experience or protection of the VHR surgical site), but if sustained, may negatively affect patient function and quality of life.^35^ Physical therapy targeted towards deficient muscle performance and disrupted kinematics effectively reduces pain and kinesiophobia,^36,37^ and improves activity par-ticipation^38^ in a variety of orthopaedic patient populations. In the proposed clinical trial, we will compare the effect of our evidence-based post-operative physical therapy program to standard of care practice (no physical therapy) in restoring abdominal core function and quality of life using both patient-reported outcomes and objective assessment of abdominal core stability. Moreover, we consider the relationships and interactions between the components of the abdominal core through the single concept of abdominal core function, which enables us to examine abdominal core health in a clinically-feasible way.^4^

# RESEARCH DESIGN AND METHODS

- 1. **Study design**

This investigation includes a two-arm registry-based randomized controlled trial comparing an evidence-based, standardized 8-week physical therapy protocol to standard of care, which only includes prescribed activity pre-cautions and the use of a binder to protect the healing construct. This registry based randomized controlled trial (RCT) will consist of 94 adult males and females between the ages of 18 and 70 who are diagnosed with ventral hernia during their initial physician evaluation and are scheduled for ventral hernia repair (VHR) within the Division of General and Gastrointestinal Surgery at the Ohio State University Wexner Medical Center (OSUWMC).

*Table 1. Subject Inclusion and Exclusion Criteria*

| **Inclusion Criteria** | **Exclusion Criteria** |
| --- | --- |
| Ages 18-70 | Previously diagnosed movement or balance disorder |
| Diagnosis of ventral hernia | Use of ambulatory assistance device (walker or cane) |
| Scheduled for elective ventral hernia repair | Not currently undergoing or planning to undergo physical therapy or other skilled exercise intervention supervised by a medical rehabilitation professional |
| Independent functional status |  |
| Transverse hernia width of 2cm or greater |  |

**Treatment Arms:**

This trial includes two post-operative treatment arms that provide clinical equipoise. Preliminary data from the ACHQC registry (unpublished) show that out of 4174 patients undergoing a VHR from inception through 7/5/2020, 91% (3805) only received post-operative precaution instructions, while 9% (369) underwent any post-operative outpatient physical therapy or a self-directed home exercise program (completed or planned) (Fig. 2A). This result clearly demonstrates that post-operative instructions without any physical therapy or exercise prescription is the standard of care among the providers contributing patients to the registry. Moreover, our preliminary data from this group shows no difference in readmission rate, re-operation rate, or surgical site infections at 30-day follow-up between those undergoing any post-operative outpatient physical therapy or self-directed exercise program vs. those undergoing the standard of care post-operative instructions (Fig. 2B). This result suggests that abdominal core rehabilitation does not create significant additional risk to the patient.

The primary endpoint of this trial is at 10-weeks post-baseline. We anticipate that participants in both groups may elect to undergo new or additional treatments after that timepoint, but those treatments will be the financial responsibility of the participant (i.e. insurance or self-pay). We will collect these data on additional treatment via participant survey and electronic health record, as rates of cross-over (control group) and continuation of PT (PT group) will inform revisions to our approach for the definitive trial.

**Control Arm:** The control arm will receive standard of care post-operative instructions typically given to patients undergoing ventral hernia repair across the world.^55^ These include limiting strenuous physical activity and a lifting restriction of no more than 10 pounds for 6 weeks. Binder use is also encouraged for 4-6 weeks after the operation.

**Abdominal Core Rehabilitation Intervention Arm (PT):** Those assigned to the intervention group will receive an evidence-based post-operative abdominal core surgery rehabilitation program delivered by physical therapists with experience in treating this patient population. The therapy protocol has been developed and standardized over 5 years through a subset of institutions within the ACHQC. It is publicly available via the ACHQC website.^3^ The overarching goal of the program is to enable painfree functional mobility and return to prior level of function through targeted exercise and movement re-education. Progression through the phases of the program is criterion-based, guided by time and patient response. The program begins with self-directed abdominal wall protection, practicing proper postures and body mechanics with daily activities, walking, and active range of motion with diaphragmatic breathing to prevent complications such as deep vein thrombosis or pneumonia (Weeks 0-2). Supervised physical therapy begins between Weeks 2-4, with goals to learn proper breathing and abdominal and pelvic floor muscle bracing techniques, use proper body mechanics with basic daily activities, and to increase walking tolerance to 30 minutes. In Weeks 4-8, the goals are to improve cardiovascular endurance, tolerate graded exposure to stress at the surgical site to promote tissue adaptation, and gradually return to normal activities of daily living. In Weeks 8+ the physical therapist guides the patient through individualized, progressive resistance training and abdominal and trunk muscle conditioning to achieve optimal muscle function. Patients with poor tolerance to land-based therapy are referred to aquatic physical therapy. In all cases, progression only proceeds when the patient can safely complete the activity on their own without increased pain; the abdomen does not significantly protrude during exercises; and the patient is able to breathe appropriately, i.e. exhale with exertion, avoiding Valsalva.^56^

Sixteen physical therapy visits (expected twice per week for 8 weeks) as needed will be paid for by the trial, so no study participants will need to pay out of pocket if they are randomized to the intervention arm. Physical therapists will follow criterion-based guidelines for exercise progression and discharge. This will allow participants who achieve acceptable outcomes prior to 16 visits to be discharged, as is consistent with ethical care practices. For participants who need additional physical therapy visits beyond what the trial can support, the participant’s insurance will be billed. The total number of visits and progress by participants through the standard protocol will be tracked for later examination (see section C.7) and will inform the rehabilitation dosage considerations for the future definitive trial.

## Identification of and Eligibility Criteria for participants

The group of patients invited to participate in this study will be ambulatory individuals who are not presenting emergently and are initially seen in an outpatient clinic and deemed suitable candidates for elective repair. The baseline study visit will occur following the standard outpatient pre-operative visit. Patients will be recruited from a busy General Surgery practice at The Ohio State Wexner Medical Center. This practice sees approximately 1,200 hernia patients yearly and performs 1,000 operations yearly on average.

## Recruitment

We will recruit 94 participants from Ohio State University’s Division of General and Gastrointestinal Surgery. Potential participants will be recruited based on the findings from their medical evaluation (see: Eligibility Criteria). This project leverages the strong clinical research infrastructure already in place at OSU and dedicated time from the Clinical Research Coordinator to fully enroll our participant sample. Our team has well-established recruitment pipelines, maintained over the last 10 years of recruitment and enrollment of participants for research in the Division of General and Gastrointestinal Surgery, which includes all patients 18 and over undergoing ventral hernia repair. In addition, OSU serves as the Data Coordination Center for the ACHQC and enrolls approximately 52 participants to the ACHQC registry monthly. Approximately 18 (35%) of the monthly ACHQC participants also meet eligibility criteria for this study. We aim to enroll 6 participants/month (i.e. 33% enrollment rate of eligible individuals), enabling full trial recruitment of 94 participants within 16 months. We allow an additional 8 months for recruiting (see 2.7 Study Timeline and 5.1 Milestone Plan for details) in case additional efforts are needed to meet demographic goals in regard to sex, race, or ethnicity. This estimate accounts for eligible individuals who may refuse based on the distance required to travel for testing and/or treatments at OSU.

## Informed consent process

The CRCs will be responsible for obtaining informed consent from participants. All CRCs within the Division of General and Gastrointestinal Surgery who will work on the study have extensive experience recruiting and enrolling adults in research studies in busy clinical settings. Patients who elect to undergo ventral hernia repair and meet all eligibility criteria will be approached by CRCs preoperatively about study participation. CRCs will discuss the study with the potential participant and provide the informed consent document for review. All potential participants will be given the opportunity to review the informed consent document in private and have the opportunity to ask questions. Potential participants will also be informed that their participation is completely voluntary and their decision to participate or not will not affect their relationship with OSUMC or their clinical care providers. They will also be informed of the potential benefits and risks, and reminded that the trial involves randomization and they have an equal chance of being assigned to either treatment group. All of this information, as well as all standard institutional elements of informed consent, will be included in the document for review and communicated verbally to the potential participant to ensure they are fully informed prior to agreeing to participate. Individuals who wish to participate in the study will complete an electronic consent form within the study’s secure REDCap^TM^ database via electronic signature.

**3.5 Randomization and Blinding**

Primary timepoints include less than 30 days pre-op (baseline) and ten weeks after surgery, with intermediate or secondary timepoints 30 days, 6 months, and 1 year post-op. On the day of surgery (immediately after the procedure), patients will be randomized (1:1) to one of two treatment arms. Randomization will be stratified by hernia width measured during the procedure using the European Hernia Society Width categories (W1 <4cm, W2 4-10cm, W3 >10cm)^53^ to ensure equitable distribution of disease severity between the two treatment arms. Since hernia width is measured during surgery, randomization will occur immediately after the procedure is completed. The study statistician will generate the randomization list using a randomized permuted block scheme. The block sizes will not be known to the Co-PIs. Study personnel involved in participant testing and the statistician will remain blinded to group assignment. Randomization will take place through the Research Electronic Data Capture (REDCap^TM^) system.^54^ Due to the nature of the two treatment arms, neither patients, treating physical therapists, or treating surgeons can be blinded to group assignment. Blinded outcomes assessment following surgery will be performed by the post-doctoral researcher. Monetary compensation will be used as an incentive to all participants for returning for the 10-week ($25), 6-month ($50), and 1-year ($100) follow-up visits.

## Data collection

## Baseline visit

The baseline visit will occur for all participants who consent to be in the study. Participants will complete the primary outcome assessments: five time sit to stand (5xSTS), and the Quiet Unstable Sitting Test (QUeST). Participants will also complete the secondary outcome assessments: PROMIS-Physical Function Computer Aided Test (PROMIS-PF-CAT), the Hernia Related Quality of Life Survey (HerQLes), the Timed Up and Go (TUG), the International Physical Activity Questionnaire – Short Form (IPAQ-SF), and the Tampa Scale of Kinesiophobia – Short Form (TSK-11). Participants in both arms will complete these assessments. The baseline assessment will normally be performed during the participant’s already-scheduled pre-operative clinic visit with the surgeon, but may be also be performed during a separate visit before the scheduled surgical procedure.

## Five Times Sit-To-Stand (5xSTS)

The 5xSTS is a common functional test recommended for falls prediction in older individuals.^48,72,73^ It is also commonly used in mobility-impaired individuals such as those with low back pain,^60^ hip osteoarthritis,^58^ Parkinson’s Disease,^74^ and stroke.^48^ Normative values for populations similar to those seeking hernia repair exist in the literature.^75^ The 5xSTS begins and ends with the participant sitting in a chair. On command, the participant rises from the chair to a full standing position and sits back down five times, as quickly as possible, without using their arms for support. Timing begins at the first movement and ends when the buttocks contact the chair after the fifth rise. In addition to timing the activity, we will record the activity using video and/or non-invasive motion sensors applied to the arms, legs, and torso to enable analysis of the movement itself. We have chosen this functional test for the hernia population because it requires bending at the waist, strength, and endurance, all challenges to the abdominal core. Moreover, in a patient with abdominal core dysfunction, we expect that an inability to rise from a chair presents significant limitations on independent mobility in activities of daily living. Our team’s preliminary data demonstrates that pre-operative hernia patients have deficient sit-to-stand function relative compared to age-matched health adults performing 5xSTS with similar instruc-tions^62^. Since 5xSTS is not included in the registry, coded 5xSTS data will be recorded and stored in a REDCap^TM^ database by the blinded tester (C.4). This primary functional outcome will be assessed by comparing the 5xSTS score at 10 weeks, 6 months, and 1 year after the operation compared to baseline (preoperative 5xSTS score).

## PROMIS-Physical Function Computer Aided Test (PROMIS-PF-CAT)

The Patient-Reported Outcomes Measurement Information System (PROMIS) was developed with funding from NIH to provide standard measures usable across a wide range of pathologies. The most relevant to our study is Physical Function (PF), and the PROMIS-PF v1.2 includes validated short form and computer-aided test (PRO-MIS-PF-CAT) versions.^63,76–78^ In orthopaedic trauma patients who can be expected to experience a wider range of physical function than hernia patients, the CAT version is extremely reliable (Cronbach ⍺ = 0.98), takes only 44 seconds to complete, and demonstrates no ceiling or floor effects.^63^ The output of the PROMIS-PF-CAT (0-100) is scaled based on the US population, where 50 represents physical function for the average adult, lower scores indicate worse physical function, and higher scores indicate better physical function. Use of PROMIS-PF is novel in abdominal surgery, with only one published study that included patients undergoing various abdominal procedures,^64^ but its responsiveness to change at one week, three weeks, and five weeks after surgery combined with its known validity in measuring physical function make it the best self-reported measure to use for this RCT. PROMIS-PF-CAT is not included in the registry, so coded PROMIS-PF-CAT data will be recorded and stored in our REDCap^TM^ database by the blinded tester (C.4). This primary self-reported outcome will be assessed by comparing the PROMIS-PF-CAT score at 10 weeks, 6 months, and 1 year after the operation com-pared to baseline (preoperative PROMIS-PF-CAT score).

**3.5.1.3 Hernia Recurrence:** Hernia recurrence will be assessed using the Hernia Recurrence Inventory (HRI). The HRI has been validated in both inguinal and ventral hernia populations to assess recurrence 1 year or greater after repair.^65,79^ Utilization of the HRI allows a patient reported outcome assessment of recurrence without involving a physical clinical visit with a physician. This secondary outcome of hernia recurrence will be assessed 1 year after the operation.

**3.5.1.4 Hernia Related Quality of Life Survey (HerQLes):** The HerQLes is an overall quality of life Likert-style questionnaire that assesses the impact of VHR on abdominal wall function, including activities of daily living, independent mobility, and mental health (Appendix 1). The survey has been validated in VHR patients and shown to improve with time after VHR as expected.^40^ The HerQLes is included in the ACHQC registry, so every consenting patient who receives a VHR at Ohio State University will have HerQLes data at multiple time points during treatment including the time points for this study without any effort needed by the research team. The 4147 VHR patients shown in Fig. 2 had a median HerQLes score of 45 pre-operatively (IQR: 23-70) and of 63 at 30 days post-op (IQR: 35-87). Preliminary data demonstrate that healthy individuals without hernia regularly achieve HerQLes scores above 90 and that VHR patients at our institution are not achieving abdominal core health and quality of life similar to their peers without hernia disease. This secondary quality of life outcome will be assessed by comparing the HerQLes summary score at 10 weeks and 1 year after the operation compared to baseline (preoperative HerQLes summary score).

**3.5.1.5 Timed Up and Go (TUG):** The TUG is a common functional test recommended by the Centers for Disease Control and Prevention,^80^and often used in mobility-impaired individuals such as those with low back pain,^81,82^ total knee arthroplasty,^83,84^ and frailty.^85–87^ Robust sets of normative values across many different populations exist.^75,88–91^ The TUG begins and ends with the participant sitting in a chair. On command, the patient rises from the chair, walks 3 meters, turns 180°, walks back, and sits back in the chair. Patients will be asked to perform this as quickly and safely as they can. It requires nothing except a chair, stopwatch, and 4 meters of open hallway, so it is easy to implement in a clinic setting. In addition to timing the activity, we will record the activity using video and/or non-invasive motion sensors applied to the arms, legs, and torso to enable analysis of the movement itself. We have chosen this functional test for the hernia population because it requires bending at the waist, walking, and turning, so it incorporates challenges to mobility both directly and indirectly attributable to the hernia repair itself. Our team’s preliminary data demonstrates that pre-operative hernia patients have deficient TUG function relative compared to age-matched health adults^67^ (Fig. 3B). Since TUG is not included in the registry, coded TUG data will be recorded and stored in a REDCap^TM^ database by the blinded tester (C.4). This secondary functional outcome will be assessed by comparing the TUG score at 10 weeks, 6 months, and 1 year after the operation compared to baseline (preoperative TUG score).

**3.5.1.6 International Physical Activity Questionnaire – Short Form (IPAQ-SF):** Physical activity level may affect recovery from hernia repair. Therefore, we plan to collect self-reported physical activity from all participants with the IPAQ-SF which includes 5 activity domains, spanning sedentary behavior and job, transportation, household and recreational activity. The IPAQ-SF is reliable with published normative data for individuals in the same age range as the target population for this trial^68^ and several studies that have used it in patients with hernia.^92–94^ We will not stratify participants in this pilot and feasibility trial based on physical activity, but we remain cognizant that this stratification may be necessary in a future definitive multi-center trial so these data will support future clinical trial design. We will consider IPAQ-SF as a potential covariate in our analyses.

**3.5.1.7 Tampa Scale of Kinesiophobia – Short Form (TSK-11):** Kinesiophobia may be a significant driver of poor physical function and quality of life common to those with hernia disease. However, it has never been measured using a standard scale in this population. Therefore, we include the TSK-11 as a measure to help us better understand the role of kinesiophobia as a covariate affecting quality of life and responsiveness to PT intervention, and consider as a candidate for inclusion in future multi-center trials. It has good reliability and responsiveness.^69^

## QUeST Core Stability Score

The QUeST is an adapted version of unstable sitting paradigms originally developed to study motor control and muscle activation patterns in individuals with low back pain by several research groups.^95–102^ These paradigms have used either a rigid hemisphere or springs mounted to the underside of the chair to make the seat unstable. In our recent NIH-funded project (R03AR065215)^4^ we adapted this paradigm to the clinic setting by using a BO-SU® trainer placed on top of a force-measuring platform (Bertec BP-5046). The participant sits quietly with eyes closed and arms crossed for 3 trials of 60s each, and counts backwards by 4’s to a 60-bpm metronome as a cognitive dual-task to reduce conscious focus on their balance. From the force platform we calculate the excursion of the center of pressure over each 60s period (CoPexc).

**3.5.1.9 Follow-Up Assessments**

Follow-up assessments will be performed by a blinded outcomes assessor at 30 days, 10 weeks, 6 months, and 1 year after the surgical procedure. All tests will be repeated at every visit. At the completion of the 10 week ($25), 6 month ($50), and 1 year ($100) follow-up assessment, the participant will be provided with an electronic gift card emailed to the address they provide. When possible, these assessments will be scheduled on the same dates as follow-up physician visits at the same clinic location, but if this is not possible they may be scheduled for different dates or locations.

- - 1. **Physical Therapy (Treatment Arm Only)**

Those assigned to the intervention group will receive an evidence-based post-operative abdominal core surgery rehabilitation program delivered by physical therapists with experience in treating this patient population. The therapy protocol has been developed and standardized over 5 years through a subset of institutions within the ACHQC. It is publicly available via the ACHQC website.^3^ The overarching goal of the program is to enable pain-free functional mobility and return to prior level of function through targeted exercise and movement re-education. Progression through the phases of the program is criterion-based, guided by time and patient response. The program begins with self-directed abdominal wall protection, practicing proper postures and body mechanics with daily activities, walking, and active range of motion with diaphragmatic breathing to prevent complications such as deep vein thrombosis or pneumonia (Weeks 0-2). Supervised physical therapy begins between Weeks 2-4, with goals to learn proper breathing and abdominal and pelvic floor muscle bracing techniques, use proper body mechanics with basic daily activities, and to increase walking tolerance to 30 minutes. In Weeks 4-8, the goals are to improve cardiovascular endurance, tolerate graded exposure to stress at the surgical site to promote tissue adaptation, and gradually return to normal activities of daily living. In Weeks 8+ the physical therapist guides the patient through individualized, progressive resistance training and abdominal and trunk muscle conditioning to achieve optimal muscle function. Patients with poor tolerance to land-based therapy are referred to aquatic physical therapy. In all cases, progression only proceeds when the patient can safely complete the activity on their own without increased pain; the abdomen does not significantly protrude during exercises; and the patient is able to breathe appropriately, i.e. exhale with exertion, avoiding Valsalva.^56^

Sixteen physical therapy visits (expected twice per week for 8 weeks) as needed will be paid for by the trial, so no study participants will need to pay out of pocket if they are randomized to the intervention arm. Physical therapists will follow criterion-based guidelines for exercise progression and discharge. This will allow participants who achieve acceptable outcomes prior to 16 visits to be discharged, as is consistent with ethical care practices. For participants who need additional physical therapy visits beyond what the trial can support, the participant’s insurance will be billed. The total number of visits and progress by participants through the standard protocol will be tracked for later examination and will inform the rehabilitation dosage considerations for the future definitive trial.

- - 1. **Study Completion**

At the end of their participation in the study, participants will be asked to complete a set of questions regarding their experience participating in the study. These questions will not be included as variables of interest in the da-ta analysis but will serve to inform the development and implementation of the future definitive trial. Questions will cover: reasons for enrollment, any perceived benefit or harm of participation, and convenience of scheduling and attending testing and PT visits (if that was their group). We will also ask whether they would recommend to a friend to participate in a similar study where individuals would be randomized to one of two types of supervised physical therapy to improve their function after VHR. Participants will be encouraged, through a free text box, to provide other information they think would be valuable to investigators.

## Potential risks

We are assessing the safety and efficacy of supervised physical therapy targeting the abdominal core following ventral hernia repair. The risks associated with undergoing ventral hernia repair (VHR) as part of this study are expected to be no different than that of patients undergoing ventral hernia repair who are not participating in research. However, to evaluate physical therapy as an appropriate and effective adjunct to VHR, we will track incidence of common post-operative complications in both treatment groups (PT vs. no PT). Surgical site occurrence requiring procedural intervention rate is 5% at 30 days;^2^ myocardial infarction rate is 0.3% at 30 days;^3^ pneumonia rate is 1% at 30 days;^3^ pulmonary embolism rate is 1% at 30 days.^3^

The risks associated with functional performance testing and physical therapy are minor and commensurate with those associated with regular exercise and/or post-operative recovery, including muscle and joint soreness, and incisional discomfort. We anticipate these may occur as a result of performing the physical performance tests (TUG, 5xSTS, QUeST) and participation in the physical therapy exercises. The testing procedures and physical therapy program implemented here at Ohio State have been well-tolerated to date and physical therapists at OSU involved in the care of these patients are aware of changes in patient status that would warrant follow-up with the patient’s surgeon.

There is also a minimal risk that the data collected from each participant may be viewed by individuals outside the research team. The risk that confidential data may be viewed is relevant to the electronic databases (EHR, ACHQC, REDCap^TM^, coded data files on the laboratory server); the risk for such a confidentiality breach is low. Patients have alternatives to participating in this randomized controlled trial. If patients refuse participation because they either (1) refuse physical therapy or (2) insist upon physical therapy following VHR, they are free to make that choice in consultation with their treating physician and will not be enrolled in the study. Their decision to enroll in the study or withdraw from the study or not will not adversely affect their relationship with OSU, their providers or their clinical care.

## Adequacy of protection against risks

## Recruitment and Informed Consent

The CRCs will be responsible for obtaining informed consent from participants. All CRCs within the Division of General and Gastrointestinal Surgery who will work on the study have extensive experience recruiting and enrolling adults in research studies in busy clinical settings. Patients who elect to undergo ventral hernia repair and meet all eligibility criteria will be approached by CRCs preoperatively about study participation. CRCs will discuss the study with the potential participant and provide the informed consent document for review. All potential participants will be given the opportunity to review the informed consent document in private and have the opportunity to ask questions. Potential participants will also be informed that their participation is completely voluntary and their decision to participate or not will not affect their relationship with OSUMC or their clinical care providers. They will also be informed of the potential benefits and risks, and reminded that the trial involves randomization and they have an equal chance of being assigned to either treatment group. All of this information, as well as all standard institutional elements of informed consent, will be included in the document for review and communicated verbally to the potential participant to ensure they are fully informed prior to agreeing to participate. Individuals who wish to participate in the study will complete an electronic consent form within the study’s secure REDCap^TM^ database via electronic signature.

## Protections Against Potential Risks

Physical therapy is commonly prescribed for individuals with painful musculoskeletal injuries of or surgeries to the spine and extremities. The risks to the participants will be minimized through several means. First, all physical therapists engaged in treating participants in this study will be trained prior to participant enrollment by Drs. Di Stasi (Co-PI) and Ward (primary treating physical therapist, see Personnel Justification for qualifications), using IRB-approved Standard Operating Procedures documents. Booster training sessions will be provided twice annually to ensure safe and standard implementation of the rehabilitation protocol. Reliability checklists will be used to ensure physical therapy documentation is accurate, and that progressions are safe and appropriate. Participants will be monitored closely for response to treatment and physical therapists will follow criteria for protocol progression.

Risks during physical therapy treatments will be minimized in the following ways:

- Participants will be provided verbal and/or written instructions regarding:
- the safe and correct technique and dosage with the exercises prescribed
- how to modify exercises and/or when to stop doing exercises
- functional activities to avoid or perform differently
- when they should contact their physician due to changes in pain or functional status
- Participants will be asked to demonstrate the exercises while the physical therapist supervises
- Pain intensity will be used to guide progression of the program, and will recorded at the beginning and end of each physical therapy session

If participants receiving physical therapy report acute onset or severe pain or sustain an acute injury they will be assessed by the treating physical therapist. If the injury episode warrants emergency medical care, the participant will be sent to the OSU’s emergency department. If the participant status warrants follow-up with Dr. Poulose, the therapist will notify the physician within 24 hours. All AE events will be shared with the non-blinded co-PIs (Di Stasi, Poulose) who will determine whether an AE occurred as a result of study participation, and communicate with the DSMB, IRB, NIDDK in accordance with their reporting guidelines.

Research team members responsible for physical performance testing will undergo training by Dr. Chaudhari (Co-PI) prior to enrolling participants, using IRB-approved Standard Operating Procedures documents. Booster training sessions will be provided twice annually to ensure safe and standard implementation of the testing protocols, including confirmation that observers achieve inter-rater and intra-rater reliability similar to published results with standardized patients.

Risks during testing will be minimized in the following ways:

- Trained research personnel will be present during all testing.
- Participants will be provided standardized verbal instructions and demonstration of all functional performance testing procedures.
- Participants will be given the opportunity to practice each task prior to testing to ensure comfort and safety.

If participants report acute onset or severe pain or sustain an acute injury during testing procedures, the participant will be immediately evaluated to determine next steps by Dr. Poulose’s (co-PI) clinical care team if tested at the physician clinic; or by Dr. Ward’s (primary treating physical therapist) clinical care team if tested at the research laboratory, which is adjacent to the physical therapy clinic.

Several methods will be used to minimize the risk that confidential data may be viewed outside of the approved study team members. All electronic data platforms used in this study (i.e. Epic EHR, ACHQC and REDCap^TM^) have several data security measures in place, including encryption, user-restricted access, and robust auditing functions. The research team is trained in undertaking precautions to limit these risks, including CITI training modules in Human Subjects Research, Responsible Conduct of Research, and Good Clinical Practice.

All PHI is maintained as part of the research participant’s medical chart (EHR), accessible only by clinical care providers and approved researchers. Consent forms will be maintained within REDCap^TM^. All electronic data exported from ACHQC and REDCap^TM^, or pulled from participants’ EHR will be securely managed behind the OSUWMC firewall and accessible only by IRB-approved personnel with usernames and passwords. Participants will not be identified in any report of the research findings.

## Potential benefits

There are no direct benefits of the proposed research to research participants and others. However, as a feasibility trial, we seek essential safety and efficacy data to either support or refute our multidisciplinary treatment approach for individuals with ventral hernia disease. Physical therapy, specifically, is not standard of care for these individuals but is commonly prescribed following lumbar spine and other orthopaedic surgeries for the lower extremity. The participants in this study benefit if the proposed therapy protocol is well-tolerated, well-attended, and improves function. The rehabilitation protocol was designed by a multicenter, multidisciplinary group of experts (i.e. surgeons, nurses and physical therapists) including members of our team at Ohio State who care for individuals with hernia disease. It includes time-and criteria-based progression of therapeutic exercises targeting abdominal care impairments and patient education to optimize function and minimize risk of post-operative complications. This trial is the first step in determining whether physical therapy is a safe and important component of patient recovery for patients undergoing VHR.

# Statistical Considerations

2.1 Sample size and Power

As indicated in the FOA (PA-20-160), this is a pilot and feasibility trial, hence it is not fully powered to answer a question. Sample size estimations are provided to demonstrate that moderate-to-large effects can be observed, but most importantly our goal is to collect a large enough sample to reasonably represent the variability in the population to power a future definitive multi-center RCT.

With 47 patients in each group, we will have 80% power to detect a 20% reduction (i.e. improvement) in 5xSTS scores at 10 weeks in the PT group compared to the control group, assuming coefficient of variation (CV)=35% and two-sided alpha of 0.025. We will also have 80% power to detect a 40% reduction in PROMIS-PF-CAT scores at 10 weeks in PT group compared to the control group, assuming CV=100% and two-sided alpha of 0.025. The overall type I error will be controlled at 0.05 using the Bonferroni method. With 47 patients in each group, we will be able to estimate the retention rates or compliance rates with 95% confidence intervals of ±11.4% if the retention or adherence rate is at least 80% in each treatment group. With 47 patients in the PT group, we will have 80% power to detect a Spearman's rank correlation of at least 0.45 between QUeST score and the change of 5xSTS / PROMIS-PF-CAT with a significance level of 0.025.

**2.2 Statistical analysis plan**

An intention to treat approach is planned for all analyses, using all participants who were randomized who have available data at the 10-week time point. Where appropriate, normality will be assessed, and non-parametric approaches will be used when assumptions are violated. Demographics, clinical characteristics (e.g. age, sex distribution, BMI, baseline pain), and all the secondary outcomes (HerQLes scores, HRI, TUG, IPAQ-SF, and TSK-11) will be summarized for the PT and control arms, respectively, and compared between the groups using two-sample t-tests for continuous outcomes or Chi-square/Fisher’s exact test for discrete outcomes. Linear mixed effect models will be used to study the effect of PT over all the time points for those outcomes with longitudinal measures, while including other covariates (biological sex, age, BMI, CDC wound class, IPAQ). To evaluate the effect of treatment group allocation on 5xSTS and the PROMIS Physical Function (Aim 1), we will use linear mixed effect models over all the time points (10 weeks, 30 days, 6 months, and 1 year). We will consider controlling for possible confounders based on the results of our group demographic comparisons. Interaction (treatment group × time) will also be tested in the model. Adjustments for multiple comparisons will be performed.

To evaluate study retention rates and treatment adherence (Aim 2) we will use descriptive statistics. We will calculate the number of participants in each treatment group who participate in testing at 10 weeks post-op (both treatment groups). Adherence rates in the PT group will be measured by session attendance, documented by the treating physical therapist. The retention rate and adherence rates will be calculated with 95% confidence intervals using exact binomials for each treatment at each follow-up. For Aim 3 we choose correlation analysis to best address the question of whether baseline abdominal core function (as measured by QUeST) indicates which patients are most likely to benefit from post-operative rehabilitation versus standard of care. As shown in Fig. 5C, some hernia patients have relatively high QUeST scores at baseline, which suggests that they may not benefit as much from physical therapy as those with lower QUeST scores.

**2.3 Safety Stopping Rules**:

As part of the DSMP for this feasibility randomized controlled trial, we will implement safety stopping rules using strict, a priori criteria, to allow trial termination due to significant harm. We do not anticipate a higher incidence of adverse events (AEs) or serious adverse events (SAEs) in our study participants than is reported in the ventral hernia repair literature due to functional performance testing or participation in physical therapy. We also do not anticipate that participants randomized to receive PT will have higher adverse event rates than those not randomized to PT. Thus, stopping the trial would require detecting a meaningful difference in these AE/SAE rates between the no PT vs PT groups.

We will monitor the rate of adverse events for each of the treatment arms (PT vs no PT) using the stopping criteria outlined below (Table 1). We will minimize the risk that patients will experience AEs in the PT arm by providing standardized physical therapist training on criterion-based exercise progressions and implementing early stopping rules (Table 1). The incidence rate of surgical site infections is 5-9% and the incidence rate of surgical site occurrence requiring procedural intervention is 5%. Therefore, we wish to ensure that the adverse events rate defined above is no greater than 15%. The following table gives stopping criteria based on Bayesian Toxicity Monitoring with a posterior probability of stopping for excessive toxicity based on current data 𝑃𝑟𝑜𝑏(𝜃>15%|𝑟,𝑛) ≥ 90%.^2^

| **Table 1. Stopping criteria based on observation of adverse events per treatment arm.** | |
| --- | --- |
| Number of participants who have begun the treatment | Halt if number of unique participants with adverse events is greater than or equal to: |
| 5 | 2 |
| 10 | 3 |
| 15 | 5 |
| 25 | 7 |
| 35 | 9 |
| 45 | 10 |

# DATA MANAGEMENT

## Data Archiving, Quality Control, and Record Retention

## Data Archiving

Research project data, forms, documents, software programs, and computer data files are indexed and archived at the time of research project completion. The index is sufficient to determine completeness of the archive. During the research project, a central index is maintained to locate primary research project documents. At the conclusion of the research project, primary research project documentation will be consolidated and retained in filling cabinets in an organized fashion, with a clear index to organization.

## Security

The databases will be maintained within the Ohio State University Wexner Medical Center firewall. Participants will be assigned unique study IDs. Any potentially identifiable information, including demographic and clinical information and videos, will be maintained on a secured study database housed behind the Medical Center firewall. Study data will only be accessed on encrypted, password-protected devices.

We will develop and implement Standard Operating Procedures (SOPs) to ensure protocol adherence, and rigorous data collection and management processes. All research personnel will receive training specific to their roles in the clinical trial by the co-PIs prior to their involvement in the study using the Manual of Operational Procedures (MOP) and SOPs. Standardized electronic templates for data input, cleaning, and export will be used and regular data audits will be performed. Clinical and survey data are collected in the Abdominal Core Health Quality Collaborative (ACHQC) national registry (i.e. HerQLes, HRI), and we will follow their current processes for data entry and export. Participants will also answer demographic questions and complete additional surveys via REDCap^TM^; the Clinical Research Coordinator will ensure surveys are complete and also use REDCap^TM^ to record functional performance testing. Unblinded Co-PI Di Stasi will perform a second data quality check of data entered into REDCap™ within 48 hours. The Ohio State University Wexner Medical Center is the ACHQC Data Coordinator Center and implements standard data assurance review procedures across ACHQC clinical sites. Both ACHQC and REDCap^TM^ maintain rigorous processes for collecting, housing, and exporting coded data. Only approved research team members will have access to these restricted data sets.

The Ohio State Wexner Medical Center is a participant of the ACHQC. The ACHQC is an organization dedicated to quality improvement in hernia and abdominal core health and already receives protected health information from this institution for routine healthcare operations under the terms of a business associate agreement. This pre-existing data collection effort will be utilized for the research study. Additional data elements, as specified in the protocol, may be combined with the ACHQC data to create a final research dataset. Registry IDs (ACHQC IDs) will be securely transmitted from The Ohio State Wexner Medical Center to the ACHQC analytic team to identify patients enrolled in the study. A curated, patient level research dataset comprised of ACHQC data (see ACHQC Data Dictionary) will be created by the ACHQC and transmitted securely to The Ohio State Wexner Medical Center. The final research dataset to be sent securely to the Sponsor will be in the form of an identifiable dataset. The specific identifiable data elements to be included in the data transfer from the ACHQC to The Ohio State Wexner Medical Center include:

ACHQC IDs, dates of service, date of birth, surgeon ID, site ID, and clinical information specified in the ACHQC Data Dictionary.

Additional research data elements will be merged with ACHQC data after the ACHQC data has been transmitted to The Ohio State Wexner Medical Center to create a final research analytic dataset. Merging of this data will be performed by the OSU and maintained securely. The data flow diagram is as follows (Sponsor = OSU):


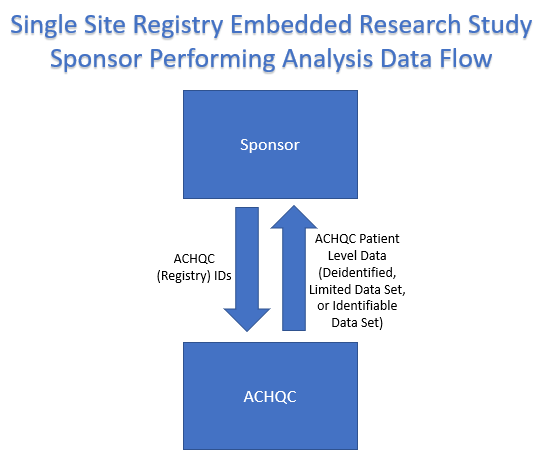


Physical therapy data will be collected through OSU’s electronic health platform (EHR), Epic. Only physical therapists who regularly see patients post-ventral hernia repair and who have been trained in trial protocol implementation will provide care for trial participants. Standardized templates will be used to record details of the exercises and education provided, allowing discrete data export for data fidelity checks. Smartphrases will be developed and approved for clinician use to ensure ease and consistency in reporting visit details, including participants’ response to and progress through the physical therapy protocol. Compliance and consistency with protocol implementation for all participants receiving physical therapy will be documented using reliability checklists. All participants’ charts will be reviewed by co-PI Di Stasi in collaboration with our primary treating physical therapist lead (Ward, see Personnel Justification for qualifications) at least once during the 10-week intervention period, ensuring data fidelity and allowing retraining and remediation for documentation processes as needed.

A number of steps will be taken to protect participant confidentiality during data collection and physical therapy treatment. Baseline testing (surveys and functional performance testing) will occur in a private treatment room. These data will be collected by trained research personnel using secure data management platforms (ACHQC and REDCap^TM^) that restrict access to approved study personnel with usernames and passwords. Each participant will have a unique identifier (UID) in the ACHQC and a separate REDCap^TM^ UID, and the correspondence between these will be kept in a separate coded sheet with no personal health information (PHI) or personally-identifiable information (PII). This will allow us to link participant data with high fidelity while maintaining data security.  Electronic health records (EHR) of all participants are maintained in Epic software, consistent with standard of care practices for individuals not involved in research. Only authorized research team members will enter participants’ EHR to gather data specific to physical therapy intervention and safety stopping rules. Access to all of these electronic platforms are restricted to authorized users only and require usernames and passwords. Certificates of Confidentiality (CoC) from the NIH will provide additional protections to participants and their data.

## Data Quality Control

## Data quality control (QC) checks will be done on a random sample of the data. A random sample of cases will be pulled and the data on the study documents will be reviewed against the data in the database. If it is determined that there are consistent errors which could potentially affect the integrity of the data, a larger sample of the block will be pulled for review. Corrective actions are determined based upon the magnitude and type of the errors found as a result of the QC.

## Record Retention

All records relating to the proposed research will be retained until all analysis has been completed, or for the length of time designated by the institutional requirements. All electronic data will be stored in a secure REDCap database until the earliest opportunity consistent with the integrity of the research.

# ETHICAL AND REGULATORY CONSIDERATIONS

## Regulatory Approval

The co-PIs and study team will obtain Institutional Review Board approval prior to the initiation of any study-related activities. If over the course of the proposed research it becomes necessary to update or amend the research as currently approved, the co-PIs will submit amendments to the Institutional Review Board and obtain approval for the new procedure(s) before any changes are enacted.

## Informed Consent

As noted in previous section(s), consent will be obtained during the preoperative period when eligible participants present to a surgical clinic. Informed consent will be obtained before any research-related interventions take place. Eligible patients will be provided ample time to review all study-related documents and ask questions of the research team as necessary. Once the eligible participant understands each section of the consent including the purpose, requirements, benefits, risks, confidentiality, right to withdraw, and contact person, then and only then will the eligible participant be asked to provide their informed consent. The eligible participant will be given a copy of the signed consent for their records.

# REFERENCES

1. Poulose BK, Shelton J, Phillips S, Moore D, Nealon W, Penson D, Beck W, Holzman MD. Epidemiology and cost of ventral hernia repair: making the case for hernia research. Hernia. 2012 Apr;16(2):179–183. PMID: 21904861

2. Poulose BK, Adrales GL, Janis JE. Abdominal Core Health-A Needed Field in Surgery. JAMA Surg. 2019 Dec 18; PMID: 31851303

3. ACHQC Abdominal Core Surgery Rehabilitation Protocol Physical Therapist Guide [Internet]. 2020 [cited 2020 Sep 10]. Available from: https://www.achqc.org/uploads/general_images/ACHQC_Abdominal_Core_Surgery_Rehabilitation_Protocol_Physical_Therapist_Guide_6.17.20.pdf

4. Chaudhari AMW, VAN Horn MR, Monfort SM, Pan X, Oñate JA, Best TM. Reducing Core Stability Influences Lower Extremity Biomechanics in Novice Runners. Med Sci Sports Exerc. 2020 Jun;52(6):1347–1353. PMCID: PMC7239732

5. PAS-20-160: Small R01s for Clinical Trials Targeting Diseases within the Mission of NIDDK (R01 Clinical Trial Required) [Internet]. [cited 2020 Sep 11]. Available from: https://grants.nih.gov/grants/guide/pa-files/PAS-20-160.html

6. Rutkow IM. Demographic and socioeconomic aspects of hernia repair in the United States in 2003. Surg Clin North Am. 2003 Oct;83(5):1045–1051, v–vi. PMID: 14533902

7. Burger JWA, Luijendijk RW, Hop WCJ, Halm JA, Verdaasdonk EGG, Jeekel J. Long-term follow-up of a randomized controlled trial of suture versus mesh repair of incisional hernia. Ann Surg. 2004 Oct;240(4):578–583; discussion 583-585. PMCID: PMC1356459

8. Luijendijk RW, Hop WC, van den Tol MP, de Lange DC, Braaksma MM, IJzermans JN, Boelhouwer RU, de Vries BC, Salu MK, Wereldsma JC, Bruijninckx CM, Jeekel J. A comparison of suture repair with mesh repair for incisional hernia. N Engl J Med. 2000 Aug 10;343(6):392–398. PMID: 10933738

9. Kokotovic D, Bisgaard T, Helgstrand F. Long-term Recurrence and Complications Associated With Elective Incisional Hernia Repair. JAMA. 2016 Oct 18;316(15):1575–1582. PMID: 27750295

10. Langbach O, Bukholm I, Benth JŠ, Røkke O. Long term recurrence, pain and patient satisfaction after ventral hernia mesh repair. World J Gastrointest Surg. 2015 Dec 27;7(12):384–393. PMCID: PMC4691719

11. Hernia Surgical Mesh Implants [Internet]. FDA; 2019 [cited 2020 May 30]. Available from: https://www.fda.gov/medical-devices/implants-and-prosthetics/hernia-surgical-mesh-implants

12. Gronnier C, Wattier J-M, Favre H, Piessen G, Mariette C. Risk factors for chronic pain after open ventral hernia repair by underlay mesh placement. World J Surg. 2012 Jul;36(7):1548–1554. PMID: 22407084

13. Hah JM, Bateman BT, Ratliff J, Curtin C, Sun E. Chronic Opioid Use After Surgery: Implications for Perioperative Management in the Face of the Opioid Epidemic. Anesth Analg. 2017 Nov;125(5):1733–1740. PMCID: PMC6119469

14. LeBlanc KA, Whitaker JM, Bellanger DE, Rhynes VK. Laparoscopic incisional and ventral hernioplasty: lessons learned from 200 patients. Hernia. 2003 Sep;7(3):118–124. PMID: 12942345

15. Heniford BT, Park A, Ramshaw BJ, Voeller G. Laparoscopic repair of ventral hernias: nine years’ experience with 850 consecutive hernias. Ann Surg. 2003 Sep;238(3):391–399; discussion 399-400. PMCID: PMC1422707

16. Haskins IN, Prabhu AS, Jensen KK, Tastaldi L, Krpata DM, Perez AJ, Tu C, Rosenblatt S, Rosen MJ. Effect of transversus abdominis release on core stability: Short-term results from a single institution. Surgery. 2019 Feb;165(2):412–416. PMID: 30224083

17. Hodges P, van den Hoorn W, Dawson A, Cholewicki J. Changes in the mechanical properties of the trunk in low back pain may be associated with recurrence. J Biomech. 2009 Jan 5;42(1):61–66. PMID: 19062020

18. Olsson A, Kiwanuka O, Wilhelmsson S, Sandblom G, Stackelberg O. Cohort study of the effect of surgical repair of symptomatic diastasis recti abdominis on abdominal trunk function and quality of life. BJS Open. 2019 Dec;3(6):750–758. PMCID: PMC6887686

19. Mehta R, Cannella M, Smith SS, Silfies SP. Altered Trunk Motor Planning in Patients with Nonspecific Low Back Pain. Journal of Motor Behavior. 2010 Jan 1;42(2):135–144. PMID: 20207604

20. Silfies SP, Mehta R, Smith SS, Karduna AR. Differences in feedforward trunk muscle activity in subgroups of patients with mechanical low back pain. Arch Phys Med Rehabil. 2009 Jul;90(7):1159–1169. PMID: 19501348

21. Silfies SP, Squillante D, Maurer P, Westcott S, Karduna AR. Trunk muscle recruitment patterns in specific chronic low back pain populations. Clin Biomech (Bristol, Avon). 2005 Jun;20(5):465–473. PMID: 15836933

22. Radebold A, Cholewicki J, Panjabi MM, Patel TC. Muscle response pattern to sudden trunk loading in healthy individuals and in patients with chronic low back pain. Spine (Phila Pa 1976). 2000 Apr 15;25(8):947–954. PMID: 10767807

23. van Dieën JH, Cholewicki J, Radebold A. Trunk muscle recruitment patterns in patients with low back pain enhance the stability of the lumbar spine. Spine (Phila Pa 1976). 2003 Apr 15;28(8):834–841. PMID: 12698129

24. van Dieën JH, Selen LPJ, Cholewicki J. Trunk muscle activation in low-back pain patients, an analysis of the literature. J Electromyogr Kinesiol. 2003 Aug;13(4):333–351. PMID: 12832164

25. Radebold A, Cholewicki J, Polzhofer GK, Greene HS. Impaired postural control of the lumbar spine is associated with delayed muscle response times in patients with chronic idiopathic low back pain. Spine (Phila Pa 1976). 2001 Apr 1;26(7):724–730. PMID: 11295888

26. Zazulak BT, Hewett TE, Reeves NP, Goldberg B, Cholewicki J. Deficits in neuromuscular control of the trunk predict knee injury risk: a prospective biomechanical-epidemiologic study. Am J Sports Med. 2007 Jul;35(7):1123–1130. PMID: 17468378

27. Salomoni SE, Marinovic W, Carroll TJ, Hodges PW. Motor Strategies Learned during Pain Are Sustained upon Pain-free Reexposure to Task. Med Sci Sports Exerc. 2019;51(11):2334–2343. PMID: 31261214

28. Summers SJ, Chipchase LS, Hirata R, Graven-Nielsen T, Cavaleri R, Schabrun SM. Motor adaptation varies between individuals in the transition to sustained pain. Pain. 2019;160(9):2115–2125. PMID: 31095094

29. Thomas JS, France CR, Lavender SA, Johnson MR. Effects of fear of movement on spine velocity and acceleration after recovery from low back pain. Spine. 2008 Mar 1;33(5):564–570. PMID: 18317203

30. Trigsted SM, Cook DB, Pickett KA, Cadmus-Bertram L, Dunn WR, Bell DR. Greater fear of reinjury is related to stiffened jump-landing biomechanics and muscle activation in women after ACL reconstruction. Knee Surg Sports Traumatol Arthrosc. 2018 Dec;26(12):3682–3689. PMID: 29700560

31. Watson PJ, Booker CK, Main CJ. Evidence for the Role of Psychological Factors in Abnormal Paraspinal Activity in Patients with Chronic Low Back Pain. Journal of Musculoskeletal Pain. 1997 Jan 1;5(4):41–56.

32. Ardern CL, Österberg A, Tagesson S, Gauffin H, Webster KE, Kvist J. The impact of psychological readiness to return to sport and recreational activities after anterior cruciate ligament reconstruction. Br J Sports Med. 2014 Dec;48(22):1613–1619. PMID: 25293342

33. Baez SE, Hoch MC, Hoch JM. Psychological factors are associated with return to pre-injury levels of sport and physical activity after ACL reconstruction. Knee Surg Sports Traumatol Arthrosc. 2020 Feb;28(2):495–501. PMID: 31486916

34. Glaws KR, Ellis TJ, Hewett TE, Di Stasi S. Return to Sport Rates in Physically Active Individuals 6 Months After Arthroscopy for Femoroacetabular Impingement Syndrome. J Sport Rehabil. 2019 Aug 1;28(6):570–575. PMID: 29651906

35. Goldberg P, Zeppieri G, Bialosky J, Bocchino C, van den Boogaard J, Tillman S, Chmielewski TL. Kinesiophobia and Its Association With Health-Related Quality of Life Across Injury Locations. Arch Phys Med Rehabil. 2018;99(1):43–48. PMID: 28760572

36. Wellsandt E, Khandha A, Manal K, Axe MJ, Buchanan TS, Snyder-Mackler L. Predictors of knee joint loading after anterior cruciate ligament reconstruction. Journal of orthopaedic research : official publication of the Orthopaedic Research Society. 2017 Mar;35(3):651–656. PMID: 27747918

37. Kernan T, Rainville J. Observed outcomes associated with a quota-based exercise approach on measures of kinesiophobia in patients with chronic low back pain. J Orthop Sports Phys Ther. 2007 Nov;37(11):679–687. PMID: 18057675

38. Aasa B, Berglund L, Michaelson P, Aasa U. Individualized low-load motor control exercises and education versus a high-load lifting exercise and education to improve activity, pain intensity, and physical performance in patients with low back pain: a randomized controlled trial. J Orthop Sports Phys Ther. 2015 Feb;45(2):77–85, B1-4. PMID: 25641309

39. Holihan JL, Hannon C, Goodenough C, Flores-Gonzalez JR, Itani KM, Olavarria O, Mo J, Ko TC, Kao LS, Liang MK. Ventral Hernia Repair: A Meta-Analysis of Randomized Controlled Trials. Surg Infect (Larchmt). 2017 Sep;18(6):647–658. PMID: 28557648

40. Krpata DM, Schmotzer BJ, Flocke S, Jin J, Blatnik JA, Ermlich B, Novitsky YW, Rosen MJ. Design and initial implementation of HerQLes: a hernia-related quality-of-life survey to assess abdominal wall function. J Am Coll Surg. 2012 Nov;215(5):635–642. PMID: 22867715

41. Poulose BK, Roll S, Murphy JW, Matthews BD, Todd Heniford B, Voeller G, Hope WW, Goldblatt MI, Adrales GL, Rosen MJ. Design and implementation of the Americas Hernia Society Quality Collaborative (AHSQC): improving value in hernia care. Hernia. 2016 Apr;20(2):177–189. PMID: 26936373

42. Itani KMF, Hur K, Kim LT, Anthony T, Berger DH, Reda D, Neumayer L, Veterans Affairs Ventral Incisional Hernia Investigators. Comparison of laparoscopic and open repair with mesh for the treatment of ventral incisional hernia: a randomized trial. Arch Surg. 2010 Apr;145(4):322–328; discussion 328. PMID: 20404280

43. Matar HE, Platt SR, Gollish JD, Cameron HU. Overview of Randomized Controlled Trials in Total Knee Arthroplasty (47,675 Patients): What Have We Learnt? J Arthroplasty. 2020 Jun;35(6):1729-1736.e1. PMID: 32088054

44. Alkhatib H, Tastaldi L, Krpata DM, Petro CC, Fafaj A, Rosenblatt S, Rosen MJ, Prabhu AS. Outcomes of transversus abdominis release (TAR) with permanent synthetic retromuscular reinforcement for bridged repairs in massive ventral hernias: a retrospective review. Hernia. 2020 Apr;24(2):341–352. PMID: 31549325

45. Conze J, Kingsnorth AN, Flament JB, Simmermacher R, Arlt G, Langer C, Schippers E, Hartley M, Schumpelick V. Randomized clinical trial comparing lightweight composite mesh with polyester or polypropylene mesh for incisional hernia repair. Br J Surg. 2005 Dec;92(12):1488–1493. PMID: 16308855

46. Bennell K, Dobson F, Hinman R. Measures of physical performance assessments: Self-Paced Walk Test (SPWT), Stair Climb Test (SCT), Six-Minute Walk Test (6MWT), Chair Stand Test (CST), Timed Up & Go (TUG), Sock Test, Lift and Carry Test (LCT), and Car Task. Arthritis Care Res (Hoboken). 2011 Nov;63 Suppl 11:S350-370. PMID: 22588756

47. Moore JL, Potter K, Blankshain K, Kaplan SL, O’Dwyer LC, Sullivan JE. A Core Set of Outcome Measures for Adults With Neurologic Conditions Undergoing Rehabilitation. J Neurol Phys Ther. 2018 Jul;42(3):174–220. PMCID: PMC6023606

48. Whitney SL, Wrisley DM, Marchetti GF, Gee MA, Redfern MS, Furman JM. Clinical measurement of sit-to-stand performance in people with balance disorders: validity of data for the Five-Times-Sit-to-Stand Test. Phys Ther. 2005 Oct;85(10):1034–1045. PMID: 16180952

49. Gautschi OP, Smoll NR, Corniola MV, Joswig H, Chau I, Hildebrandt G, Schaller K, Stienen MN. Validity and Reliability of a Measurement of Objective Functional Impairment in Lumbar Degenerative Disc Disease: The Timed Up and Go (TUG) Test. Neurosurgery. 2016 Aug;79(2):270–278. PMID: 26702840

50. Barry E, Galvin R, Keogh C, Horgan F, Fahey T. Is the Timed Up and Go test a useful predictor of risk of falls in community dwelling older adults: a systematic review and meta-analysis. BMC Geriatr. 2014 Feb 1;14:14. PMCID: PMC3924230

51. Fröbert O, Lagerqvist B, Olivecrona GK, Omerovic E, Gudnason T, Maeng M, Aasa M, Angerås O, Calais F, Danielewicz M, Erlinge D, Hellsten L, Jensen U, Johansson AC, Kåregren A, Nilsson J, Robertson L, Sandhall L, Sjögren I, Ostlund O, Harnek J, James SK, TASTE Trial. Thrombus aspiration during ST-segment elevation myocardial infarction. N Engl J Med. 2013 Oct 24;369(17):1587–1597. PMID: 23991656

52. Lauer MS, D’Agostino RB. The randomized registry trial--the next disruptive technology in clinical research? N Engl J Med. 2013 Oct 24;369(17):1579–1581. PMID: 23991657

53. Muysoms FE, Miserez M, Berrevoet F, Campanelli G, Champault GG, Chelala E, Dietz UA, Eker HH, El Nakadi I, Hauters P, Hidalgo Pascual M, Hoeferlin A, Klinge U, Montgomery A, Simmermacher RKJ, Simons MP, Smietański M, Sommeling C, Tollens T, Vierendeels T, Kingsnorth A. Classification of primary and incisional abdominal wall hernias. Hernia. 2009 Aug;13(4):407–414. PMCID: PMC2719726

54. Harris PA, Taylor R, Thielke R, Payne J, Gonzalez N, Conde JG. Research electronic data capture (REDCap)--a metadata-driven methodology and workflow process for providing translational research informatics support. J Biomed Inform. 2009 Apr;42(2):377–381. PMCID: PMC2700030

55. Operation Brochures for Patients: Adult Ventral Hernia Repair [Internet]. American College of Surgeons. 2017 [cited 2021 Jan 22]. Available from: https://www.facs.org/-/media/files/education/patient-ed/ventral_hernia.ashx

56. Porth CJ, Bamrah VS, Tristani FE, Smith JJ. The Valsalva maneuver: mechanisms and clinical implications. Heart Lung. 1984 Sep;13(5):507–518. PMID: 6565684

57. Goldberg A, Chavis M, Watkins J, Wilson T. The five-times-sit-to-stand test: validity, reliability and detectable change in older females. Aging Clin Exp Res. 2012 Aug;24(4):339–344. PMID: 23238309

58. Lin YC, Davey RC, Cochrane T. Tests for physical function of the elderly with knee and hip osteoarthritis. Scand J Med Sci Sports. 2001 Oct;11(5):280–286. PMID: 11696212

59. Bohannon RW. Test-retest reliability of the five-repetition sit-to-stand test: a systematic review of the literature involving adults. J Strength Cond Res. 2011 Nov;25(11):3205–3207. PMID: 21904240

60. Smeets RJEM, Hijdra HJM, Kester ADM, Hitters MWGC, Knottnerus JA. The usability of six physical performance tasks in a rehabilitation population with chronic low back pain. Clin Rehabil. 2006 Nov;20(11):989–997. PMID: 17065542

61. Meretta BM, Whitney SL, Marchetti GF, Sparto PJ, Muirhead RJ. The five times sit to stand test: responsiveness to change and concurrent validity in adults undergoing vestibular rehabilitation. J Vestib Res. 2006;16(4–5):233–243. PMID: 17538213

62. Isles RC, Choy NLL, Steer M, Nitz JC. Normal values of balance tests in women aged 20-80. J Am Geriatr Soc. 2004 Aug;52(8):1367–1372. PMID: 15271128

63. Hung M, Stuart AR, Higgins TF, Saltzman CL, Kubiak EN. Computerized Adaptive Testing Using the PROMIS Physical Function Item Bank Reduces Test Burden With Less Ceiling Effects Compared With the Short Musculoskeletal Function Assessment in Orthopaedic Trauma Patients. J Orthop Trauma. 2014 Aug;28(8):439–443. PMID: 24378399

64. van der Meij E, Anema JR, Huirne JAF, Terwee CB. Using PROMIS for measuring recovery after abdominal surgery: a pilot study. BMC Health Serv Res. 2018 Feb 20;18(1):128. PMCID: PMC5819257

65. Baucom RB, Ousley J, Feurer ID, Beveridge GB, Pierce RA, Holzman MD, Sharp KW, Poulose BK. Patient reported outcomes after incisional hernia repair-establishing the ventral hernia recurrence inventory. Am J Surg. 2016 Jul;212(1):81–88. PMID: 26319337

66. Gautschi OP, Stienen MN, Corniola MV, Joswig H, Schaller K, Hildebrandt G, Smoll NR. Assessment of the Minimum Clinically Important Difference in the Timed Up and Go Test After Surgery for Lumbar Degenerative Disc Disease. Neurosurgery. 2017 Mar 1;80(3):380–385. PMID: 27352275

67. Bohannon RW, Bubela DJ, Magasi SR, Wang Y-C, Gershon RC. Sit-to-stand test: Performance and determinants across the age-span. Isokinet Exerc Sci. 2010;18(4):235–240. PMCID: PMC4293702

68. Craig CL, Marshall AL, Sjöström M, Bauman AE, Booth ML, Ainsworth BE, Pratt M, Ekelund U, Yngve A, Sallis JF, Oja P. International physical activity questionnaire: 12-country reliability and validity. Med Sci Sports Exerc. 2003 Aug;35(8):1381–1395. PMID: 12900694

69. Woby SR, Roach NK, Urmston M, Watson PJ. Psychometric properties of the TSK-11: a shortened version of the Tampa Scale for Kinesiophobia. Pain. 2005 Sep;117(1–2):137–144. PMID: 16055269

70. Hartigan EH, Lynch AD, Logerstedt DS, Chmielewski TL, Snyder-Mackler L. Kinesiophobia after anterior cruciate ligament rupture and reconstruction: noncopers versus potential copers. J Orthop Sports Phys Ther. 2013 Nov;43(11):821–832. PMCID: PMC4915102

71. Sánchez-Herán Á, Agudo-Carmona D, Ferrer-Peña R, López-de-Uralde-Villanueva I, Gil-Martínez A, Paris-Alemany A, La Touche R. Postural Stability in Osteoarthritis of the Knee and Hip: Analysis of Association With Pain Catastrophizing and Fear-Avoidance Beliefs. PM R. 2016 Jul;8(7):618–628. PMID: 26578431

72. Zhang F, Ferrucci L, Culham E, Metter EJ, Guralnik J, Deshpande N. Performance on five times sit-to-stand task as a predictor of subsequent falls and disability in older persons. J Aging Health. 2013 Apr;25(3):478–492. PMID: 23407343

73. Buatois S, Miljkovic D, Manckoundia P, Gueguen R, Miget P, Vançon G, Perrin P, Benetos A. Five times sit to stand test is a predictor of recurrent falls in healthy community-living subjects aged 65 and older. J Am Geriatr Soc. 2008 Aug;56(8):1575–1577. PMID: 18808608

74. Duncan RP, Leddy AL, Earhart GM. Five times sit-to-stand test performance in Parkinson’s disease. Arch Phys Med Rehabil. 2011 Sep;92(9):1431–1436. PMCID: PMC3250986

75. Bohannon RW. Reference values for the timed up and go test: a descriptive meta-analysis. J Geriatr Phys Ther. 2006;29(2):64–68. PMID: 16914068

76. Brodke DS, Goz V, Voss MW, Lawrence BD, Spiker WR, Hung M. PROMIS® PF CAT Outperforms the ODI and SF-36 Physical Function Domain in Spine Patients. Spine (Phila Pa 1976). 2017 Jun 15;42(12):921–929. PMCID: PMC5408297

77. Rose M, Bjorner JB, Becker J, Fries JF, Ware JE. Evaluation of a preliminary physical function item bank supported the expected advantages of the Patient-Reported Outcomes Measurement Information System (PROMIS). J Clin Epidemiol. 2008 Jan;61(1):17–33. PMID: 18083459

78. DeWalt DA, Rothrock N, Yount S, Stone AA. Evaluation of Item Candidates: The PROMIS Qualitative Item Review. Med Care. 2007 May;45(5 Suppl 1):S12–S21. PMCID: PMC2810630

79. Tastaldi L, Barros PHF, Krpata DM, Prabhu AS, Rosenblatt S, Petro CC, Alkhatib H, Szutan LA, Silva RA, Olson MA, Stewart TG, Roll S, Rosen MJ, Poulose BK. Hernia recurrence inventory: inguinal hernia recurrence can be accurately assessed using patient-reported outcomes. Hernia. 2020 Feb;24(1):127–135. PMID: 31359209

80. Materials for Healthcare Providers | STEADI - Older Adult Fall Prevention | CDC Injury Center [Internet]. 2020 [cited 2020 Sep 16]. Available from: https://www.cdc.gov/steadi/materials.html

81. Cruz-Díaz D, Martínez-Amat A, De la Torre-Cruz MJ, Casuso RA, de Guevara NML, Hita-Contreras F. Effects of a six-week Pilates intervention on balance and fear of falling in women aged over 65 with chronic low-back pain: A randomized controlled trial. Maturitas. 2015 Dec;82(4):371–376. PMID: 26277254

82. Coyle PC, Velasco T, Sions JM, Hicks GE. Lumbar Mobility and Performance-Based Function: An Investigation in Older Adults with and without Chronic Low Back Pain. Pain Med. 2017 Jan 1;18(1):161–168. PMCID: PMC5283703

83. Boonstra MC, De Waal Malefijt MC, Verdonschot N. How to quantify knee function after total knee arthroplasty? Knee. 2008 Oct;15(5):390–395. PMID: 18620863

84. Freter SH, Fruchter N. Relationship between timed “up and go” and gait time in an elderly orthopaedic rehabilitation population. Clin Rehabil. 2000 Feb;14(1):96–101. PMID: 10688350

85. van Iersel MB, Munneke M, Esselink RAJ, Benraad CEM, Olde Rikkert MGM. Gait velocity and the Timed-Up-and-Go test were sensitive to changes in mobility in frail elderly patients. J Clin Epidemiol. 2008 Feb;61(2):186–191. PMID: 18177792

86. Savva GM, Donoghue OA, Horgan F, O’Regan C, Cronin H, Kenny RA. Using timed up-and-go to identify frail members of the older population. J Gerontol A Biol Sci Med Sci. 2013 Apr;68(4):441–446. PMID: 22987796

87. Podsiadlo D, Richardson S. The timed “Up & Go”: a test of basic functional mobility for frail elderly persons. J Am Geriatr Soc. 1991 Feb;39(2):142–148. PMID: 1991946

88. Ibrahim A, Singh DKA, Shahar S. “Timed Up and Go” test: Age, gender and cognitive impairment stratified normative values of older adults. PLoS One. 2017;12(10):e0185641. PMCID: PMC5626462

89. Pondal M, del Ser T. Normative data and determinants for the timed “up and go” test in a population-based sample of elderly individuals without gait disturbances. J Geriatr Phys Ther. 2008;31(2):57–63. PMID: 19856551

90. Kear BM, Guck TP, McGaha AL. Timed Up and Go (TUG) Test: Normative Reference Values for Ages 20 to 59 Years and Relationships With Physical and Mental Health Risk Factors. J Prim Care Community Health. 2017 Jan;8(1):9–13. PMCID: PMC5932649

91. Kenny RA, Coen RF, Frewen J, Donoghue OA, Cronin H, Savva GM. Normative values of cognitive and physical function in older adults: findings from the Irish Longitudinal Study on Ageing. J Am Geriatr Soc. 2013 May;61 Suppl 2:S279-290. PMID: 23662720

92. Gunnarsson U, Johansson M, Strigård K. Assessment of abdominal muscle function using the Biodex System-4. Validity and reliability in healthy volunteers and patients with giant ventral hernia. Hernia. 2011 Aug;15(4):417–421. PMID: 21380564

93. Jensen KK, Kjaer M, Jorgensen LN. Isometric abdominal wall muscle strength assessment in individuals with incisional hernia: a prospective reliability study. Hernia. 2016 Dec;20(6):831–837. PMID: 27169719

94. Jensen KK, Arnesen RB, Christensen JK, Bisgaard T, Jørgensen LN. Large Incisional Hernias Increase in Size. J Surg Res. 2019 Dec;244:160–165. PMID: 31295649

95. Silfies SP, Cholewicki J, Radebold A. The effects of visual input on postural control of the lumbar spine in unstable sitting. Hum Mov Sci. 2003 Aug;22(3):237–252. PMID: 12967756

96. Butowicz CM, Ebaugh DD, Noehren B, Silfies SP. Validation of Two Clinical Measures Of Core Stability. Int J Sports Phys Ther. 2016 Feb;11(1):15–23. PMCID: PMC4739044

97. Barbado D, Moreside J, Vera-Garcia FJ. Reliability and Repetition Effect of the Center of Pressure and Kinematics Parameters That Characterize Trunk Postural Control During Unstable Sitting Test. PM R. 2017 Mar;9(3):219–230. PMID: 27616542

98. Cholewicki J, Polzhofer GK, Radebold A. Postural control of trunk during unstable sitting. J Biomech. 2000 Dec;33(12):1733–1737. PMID: 11006402

99. Larivière C, Mecheri H, Shahvarpour A, Gagnon D, Shirazi-Adl A. Criterion validity and between-day reliability of an inertial-sensor-based trunk postural stability test during unstable sitting. J Electromyogr Kinesiol. 2013 Aug;23(4):899–907. PMID: 23582401

100. Lee H, Granata KP, Madigan ML. Effects of trunk exertion force and direction on postural control of the trunk during unstable sitting. Clin Biomech (Bristol, Avon). 2008 Jun;23(5):505–509. PMID: 18282647

101. Preuss RA, Grenier SG, McGill SM. Postural control of the lumbar spine in unstable sitting. Arch Phys Med Rehabil. 2005 Dec;86(12):2309–2315. PMID: 16344028

102. van Dieën JH, Koppes LLJ, Twisk JWR. Low back pain history and postural sway in unstable sitting. Spine (Phila Pa 1976). 2010 Apr 1;35(7):812–817. PMID: 20195213

103. Maurer C, Peterka RJ. A new interpretation of spontaneous sway measures based on a simple model of human postural control. J Neurophysiol. 2005 Jan;93(1):189–200. PMID: 15331614

104. Turolla A, Rossettini G, Viceconti A, Palese A, Geri T. Musculoskeletal Physical Therapy During the COVID-19 Pandemic: Is Telerehabilitation the Answer? Phys Ther. 2020 Aug 12;100(8):1260–1264. PMCID: PMC7239136

105. Lee AC. COVID-19 and the Advancement of Digital Physical Therapist Practice and Telehealth. Phys Ther. 2020 Jul 19;100(7):1054–1057. PMCID: PMC7197535

106. Olayiwola JN, Magaña C, Harmon A, Nair S, Esposito E, Harsh C, Forrest LA, Wexler R. Telehealth as a Bright Spot of the COVID-19 Pandemic: Recommendations From the Virtual Frontlines (“Frontweb”). JMIR Public Health Surveill [Internet]. 2020 Jun 25 [cited 2021 Jan 30];6(2). Available from: https://www.ncbi.nlm.nih.gov/pmc/articles/PMC7318864/ PMCID: PMC7318864

107. Zolin SJ, Petro CC, Prabhu AS, Fafaj A, Thomas JD, Horne CM, Tastaldi L, Alkhatib H, Krpata DM, Rosenblatt S, Rosen MJ. Registry-Based Randomized Controlled Trials: A New Paradigm for Surgical Research. Journal of Surgical Research. Elsevier; 2020 Nov 1;255:428–435. PMID: 32619857
