# Supplementary material for: ABVENTURE-P pilot trial of physical therapy versus standard of care following ventral hernia repair: Protocol for a randomized controlled trial
Source: PLoS One. 2023 Jul 27;18(7):e0289038. doi: 10.1371/journal.pone.0289038 (PMC10374060; doi:10.1371/journal.pone.0289038)
Supplement: Attachment — Submitted filename: Response to Reviewers.docx [file pone.0289038.s004.docx]

*1. Please ensure that your manuscript meets PLOS ONE's style requirements, including those for file naming. The PLOS ONE style templates can be found at*

[*https://journals.plos.org/plosone/s/file?id=wjVg/PLOSOne_formatting_sample_main_body.pdf*](https://urldefense.com/v3/__https:/journals.plos.org/plosone/s/file?id=wjVg*PLOSOne_formatting_sample_main_body.pdf__;Lw!!KGKeukY!xnHwc52yBISoWl6b2s3IbQU6zi_5zrRboiy0IAlDxpyhMI-SUI5lGc2wn8lcwsSiUSteGmFvHOGJ62mw5fo$) *and*

[*https://journals.plos.org/plosone/s/file?id=ba62/PLOSOne_formatting_sample_title_authors_affiliations.pdf*](https://urldefense.com/v3/__https:/journals.plos.org/plosone/s/file?id=ba62*PLOSOne_formatting_sample_title_authors_affiliations.pdf__;Lw!!KGKeukY!xnHwc52yBISoWl6b2s3IbQU6zi_5zrRboiy0IAlDxpyhMI-SUI5lGc2wn8lcwsSiUSteGmFvHOGJF4wnTj0$)

**Response:** Thank you for the PDF instructions, the file names and manuscript formatting are now in line with the PLOS ONE style requirement templates.

*2. Thank you for stating the following in the Acknowledgments Section of your manuscript: “This study is supported by funding from the National Institute of Diabetes and Digestive and Kidney Diseases (NIDDK) (R01DK131207)[56]. This project also receives in-kind administrative support by The Ohio State University Center for Clinical and Translational Science, which receives financial support from the National Center for Advancing Translational Sciences (NCATS) (UL1TR002733) and by the Abdominal Core Health Quality Collaborative (ACHQC). Neither NIDDK nor NCATS assisted in the design of the trial and will not contribute to data collection and management, analysis and interpretation, dissemination of trial findings, and does not have authority over these activities. The content is solely the responsibility of the authors and does not necessarily represent the official views of the National Institutes of Health or the ACHQC.” We note that you have provided funding information that is currently declared in your Funding Statement. However, funding information should not appear in the Acknowledgments section or other areas of your manuscript. We will only publish funding information present in the Funding Statement section of the online submission form.*

*Please remove any funding-related text from the manuscript and let us know how you would like to update your Funding Statement. Currently, your Funding Statement reads as follows:*

*“This study is funded by the National Institute of Diabetes, Digestive and Kidney Diseases (*[*https://www.niddk.nih.gov*](https://urldefense.com/v3/__https:/www.niddk.nih.gov__;!!KGKeukY!xnHwc52yBISoWl6b2s3IbQU6zi_5zrRboiy0IAlDxpyhMI-SUI5lGc2wn8lcwsSiUSteGmFvHOGJ1wlxdf8$)*) grant R01DK131207 (SD, AMWC, BKP MPI's). This project also receives in-kind administrative support by The Ohio State University Center for Clinical and Translational Science, which receives financial support from the National Center for Advancing Translational Sciences (NCATS) (UL1TR002733) and by the Abdominal Core Health Quality Collaborative (ACHQC). Neither NIDDK nor NCATS assisted in the design of the trial and will not contribute to data collection and management, analysis and interpretation, dissemination of trial findings, and does not have authority over these activities. The content is solely the responsibility of the authors and does not necessarily represent the official views of the National Institutes of Health. The funders had and will not have a role in study design, data collection and analysis, decision to publish, or preparation of the manuscript.”*

*Please include your amended statements within your cover letter; we will change the online submission form on your behalf.*

**Response:** We have included the amended funding statement in the cover letter and deleted it from the Acknowledgments section. In the acknowledgements section, we added:

We would like to express our sincere gratitude to the physical therapists from the Sports Medicine and Ambulatory physical therapy clinics at the Ohio State University Wexner Medical Center (OSUWMC) who volunteered to serve as interventionists on this trial. We value their expertise and willingness to spend extra time completing required training to work with our participants. We also acknowledge the enthusiasm and commitment of the participating surgeons at OSUWMC, whose collaboration with our clinical research team members (Kiana Shannon and Mahsa Adib) will ensure that interested and eligible patients have the opportunity to participate in this study.

*3. Thank you for stating the following in the Competing Interests section:*

*“I have read the journal's policy and the authors of this manuscript have the following competing interests: Benjamin K. Poulose receives salary support as Vice President of the Board of the Abdominal Core Health Quality Collaborative and is an equity holder in EndoEvolve, LLC.”*

*Please confirm that this does not alter your adherence to all PLOS ONE policies on sharing data and materials, by including the following statement: "This does not alter our adherence to  PLOS ONE policies on sharing data and materials.” (as detailed online in our guide for authors* [*http://journals.plos.org/plosone/s/competing-interests).*](https://urldefense.com/v3/__http:/journals.plos.org/plosone/s/competing-interests).__;!!KGKeukY!xnHwc52yBISoWl6b2s3IbQU6zi_5zrRboiy0IAlDxpyhMI-SUI5lGc2wn8lcwsSiUSteGmFvHOGJAKN5B1k$)  *If there are restrictions on sharing of data and/or materials, please state these. Please note that we cannot proceed with consideration of your article until this information has been declared.*

*Please include your updated Competing Interests statement in your cover letter; we will change the online submission form on your behalf.*

**Response:** We conform that there is no change in adherence to the PLOS ONE policies on sharing data and materials. We have added the statement to our Competing Interests statement and put it in the cover letter.

**In-text changes:** I have read the journal's policy and the authors of this manuscript have the following competing interests: Benjamin K. Poulose receives salary support as Vice President of the Board of the Abdominal Core Health Quality Collaborative and is an equity holder in EndoEvolve, LLC. This does not alter our adherence to  PLOS ONE policies on sharing data and materials.

*4. Please amend either the title on the online submission form (via Edit Submission) or the title in the manuscript so that they are identical.*

**Response:** Thank you for noticing this. We have amended the title in the online submission form to match the title in the manuscript.

*5. Please upload a copy of Figure 5, to which you refer in your text on page 14. If the figure is no longer to be included as part of the submission please remove all reference to it within the text.*

**Response**: Thank you for noticing this. There is no Figure 5 to refer to, so we have updated the text to remove all references to Figure 5.

*6. Please review your reference list to ensure that it is complete and correct. If you have cited papers that have been retracted, please include the rationale for doing so in the manuscript text, or remove these references and replace them with relevant current references. Any changes to the reference list should be mentioned in the rebuttal letter that accompanies your revised manuscript. If you need to cite a retracted article, indicate the article’s retracted status in the References list and also include a citation and full reference for the retraction notice.*

**Response:** None of the papers cited have been retracted. We updated the reference list to include one new reference in the introduction in accordance with the requested changes from Reviewer #2. That reference is:

Luijendijk RW, Hop WCJ, van den Tol MP, et al. A comparison of suture repair with mesh repair for incisional hernia. N Engl J Med. 2000;343(6):392-398. doi:10.1056/NEJM200008103430603.

*Additional Editor Comments:

Authors should follow reviewer requests as soon as possible.


[Note: HTML markup is below. Please do not edit.]

Reviewers' comments:

Reviewer's Responses to Questions*

***Comments to the Author***

*Review Comments to the Author

Please use the space provided to explain your answers to the questions above and, if applicable, provide comments about issues authors must address before this protocol can be accepted for publication. You may also include additional comments for the author, including concerns about research or publication ethics.

You may also provide optional suggestions and comments to authors that they might find helpful in planning their study.


(Please upload your review as an attachment if it exceeds 20,000 characters)*

*Reviewer #1: This is a well-written protocol of a feasibility phase I/II trial of physical therapy versus standard of care for hernia. Randomization procedures are clearly specified and are well thought out. Goals of the study are clear and achievable. Statistical analysis plan is adequate. I have some comments:*

*1. What about the use of APPS to report daily symptoms and analyze this as a continuous time series?*

**Response:** Thank you for your review of our protocol paper. We value your suggestion of using an app to track daily symptoms and agree this would be very valuable information. We will consider this and other ways to monitor symptoms remotely when planning the next multi-center trial, however this is outside of the scope of the current study.

*2. Your goal is feasibility, determination of sample size for a larger study, preliminary efficacy on some metrics. You should have a separate section on what specific measures and criteria will be used to make a decision about moving to a larger study, and to specifically inform the design of such a study.*

**Response:** We have included a section at the end of the discussion to address this point.

**In text revision lines 411-417:** Data from this trial will establish whether abdominal core function predicts responsiveness to PT and provide robust preliminary data on all primary variables of interest (Table 2) in patients undergoing ventral hernia repair. Using these preliminary data, we will be able to choose the most appropriate variables based both on relevance in the population and feasibility of collection in busy clinical practice to accurately estimate needed sample sizes for the multi-center randomized control trial. Further, data from this trial will establish a detailed blueprint for collaboration between surgery and physical therapy to successfully execute the planned multi-center clinical trial.

*3. Limitations and how to handle stuff that doesn't work out should be added to the Discussion.*

**Response:** Thank you for this point, we have added a paragraph to the Discussion detailing key limitations and the plan to address these limitations.

**In-text revision lines 397-405:** There are some limitations and potential pitfalls to consider with the study design. The most relevant pitfall is inadequate recruitment of participants. To avoid this, we have chosen the inclusion criteria based on a historical volume of patients to ensure we will meet recruitment targets. We have also incentivized the study with physical therapy provided at no cost to participants and gift card compensations at the 10-week, 6-month, and 1-year data collection timepoints. Another pitfall could be loss to follow-up, however there is a plan in place to minimize this with patient incentives via gift card compensations and an intent-to-treat analysis. Similarly, there may be failure to comply with the physical therapy program. We will use an intent-to-treat analysis to address this pitfall and will report the number of physical therapy sessions attended as part of our aim to assess feasibility. Providing physical therapy at no cost to the participants is also intended to reduce non-compliance.

*Reviewer #2: Thank you for the opportunity to review this manuscript. Some points need to be clarified better. Are they:

1. Regarding the introduction, the current state of evidence on the subject of the study needs to be better defined and outlined.*

**Response:** The current state of evidence given recurrence rates from surgeries and the need to rehabilitation have been added to the first paragraph of the introduction.

**In text revision lines 49-53:** Recurrence rates for ventral hernia repair are as high as 32%, increasing both health care related costs to patients and risk of a transition to chronic post-operative pain. Given the high percentage of hernia recurrences after surgery, as well as the current opioid epidemic faced by the United States, there is a significant need for drug free treatments targeting abdominal core health to achieve optimal outcomes after ventral hernia repair.

*2. About the inclusion criteria: How will the diagnosis of ventral hernia be made?*

**Response**: The diagnosis of ventral hernia is made during their evaluation visit with their physician. This is included in text in line 120, ‘….who are diagnosed with ventral hernia during their initial physician evaluation.’ The diagnosis method is up to the discretion of the provider. We have added some examples of how these can be diagnosed in text.

**In text revision line 121:** ‘Ninety-four individuals between the ages of 18 and 70 who are diagnosed with ventral hernia during their initial physician evaluation (e.g., through physical examination, CT scans, ultrasounds) and are scheduled for ventral hernia repair (VHR) within…’

*3. On the description of interventions: Interventions can be better described and defined. They are understandable. However, as it is a protocol, these can be better described.*

**Response**: We agree that the intervention is an important component of the protocol paper, and provided a reference to the publicly available standardized protocol on the ACHQC website (reference #7 in text) as well as an Appendix that includes sample exercises, tips to elicit proper technique, dosage parameters, and criteria for progression or regression (Line 185-188). We have also provided a concise in-text overview of the intervention in lines 190-208.
